# Novel Sex Cells and Evidence for Sex Pheromones in Diatoms

**DOI:** 10.1371/journal.pone.0026923

**Published:** 2011-10-26

**Authors:** Shinya Sato, Gordon Beakes, Masahiko Idei, Tamotsu Nagumo, David G. Mann

**Affiliations:** 1 Royal Botanic Garden Edinburgh, Edinburgh, United Kingdom; 2 School of Biological Sciences, Institute of Molecular Plant Sciences, University of Edinburgh, Edinburgh, United Kingdom; 3 School of Biology, Newcastle University, Newcastle upon Tyne, United Kingdom; 4 Department of Biology, Bunkyo University, Koshigaya, Saitama, Japan; 5 Department of Biology, The Nippon Dental University, Chiyoda-ku, Tokyo, Japan; Clermont Université, France

## Abstract

**Background:**

Diatoms belong to the stramenopiles, one of the largest groups of eukaryotes, which are primarily characterized by a presence of an anterior flagellum with tubular mastigonemes and usually a second, smooth flagellum. Based on cell wall morphology, diatoms have historically been divided into centrics and pennates, of which only the former have flagella and only on the sperm. Molecular phylogenies show the pennates to have evolved from among the centrics. However, the timing of flagellum loss – whether before the evolution of the pennate lineage or after – is unknown, because sexual reproduction has been so little studied in the ‘araphid’ basal pennate lineages, to which *Pseudostaurosira* belongs.

**Methods/Principal Finding:**

Sexual reproduction of an araphid pennate, *Pseudostaurosira trainorii*, was studied with light microscopy (including time lapse observations and immunofluorescence staining observed under confocal scanning laser microscopy) and SEM. We show that the species produces motile male gametes. Motility is mostly associated with the extrusion and retrieval of microtubule-based ‘threads’, which are structures hitherto unknown in stramenopiles, their number varying from one to three per cell. We also report experimental evidence for sex pheromones that reciprocally stimulate sexualization of compatible clones and orientate motility of the male gametes after an initial ‘random walk’.

**Conclusions/Significance:**

The threads superficially resemble flagella, in that both are produced by male gametes and contain microtubules. However, one striking difference is that threads cannot beat or undulate and have no motility of their own, and they do not bear mastigonemes. Threads are sticky and catch and draw objects, including eggs. The motility conferred by the threads is probably crucial for sexual reproduction of *P. trainorii*, because this diatom is non-motile in its vegetative stage but obligately outbreeding. Our pheromone experiments are the first studies in which gametogenesis has been induced in diatoms by cell-free exudates, opening new possibilities for molecular ‘dissection’ of sexualization.

## Introduction

The morphology of flagella and their associated apparatus is for the most part highly conserved across each of the major groups of eukaryotes (e.g. [1]), despite huge variation in life form and life cycle. For example, the stramenopiles are a highly diverse assemblage of photosynthetic, parasitic and saprophytic organisms, including unicellular and multicellular forms [2], [3], [4], but the flagellate cells are of a characteristic type, being biflagellate and possessing a longer anterior flagellum with tubular mastigonemes, which produces a backward current to create thrust, and a shorter posterior flagella used as a rudder [5], [6], [7]. However, there are exceptions, notably in the diatoms, where only some lineages possess flagella and then only in the male gametes; furthermore these sperm have only one flagellum and even this is atypical, lacking the central pair of microtubules, i.e. there is a 9 + 0 microtubular configuration in the axoneme [8]. No posterior flagellum, not even a rudimentary one, has been found in any diatom.

Historically, diatoms have been divided into two groups, centrics and pennates, based primarily on their cell wall morphology. The pennates were further subdivided into non-motile araphid and motile raphid forms [9]. Molecular phylogenies have indicated, however, that in the pennate clade the monophyletic raphids diverged from among the araphids, making the araphids paraphyletic (reviewed by [10], [11], [12]). With respect to sexual reproduction, all the centrics (also a paraphyletic assemblage) are oogamous, producing eggs and uniflagellate sperms, whereas pennates are generally morphologically isogamous and non-flagellate [13], [14]. As an exceptional case, two araphid genera are known to exhibit anisogamy, in which compatible clones are differentiated into female and male and produce sessile eggs and smaller amoeboid male gametes, respectively [15], [16], but the latter appear to be entirely without flagella. However, the timing of flagellum loss – whether it occurred before the evolution of the pennate lineage or after – is unknown, because sexual reproduction has been so little studied in the critical basal lineages of pennates.

During a study of heterothallic sexual reproduction in an araphid pennate, *Pseudostaurosira trainorii* E. Morales, we found that male clones produce motile gametes. Further observation revealed that the gametes bear thread-like structures, which superficially resemble flagella. Here we report the sexual reproduction of *P. trainorii* with special emphasis on the behaviour and fine structure of the threads, examined using light microscopy (LM), including time lapse observations; immunofluorescence staining observed under confocal scanning laser microscopy (CSLM) and scanning electron microscopy (SEM). We demonstrate that the threads do not correspond to flagella in morphology, structure or activity. We also report experimental evidence for the presence of sex pheromones that reciprocally stimulate sexualization of compatible clones.

## Results

### Vegetative cells

Cells were rectangular in girdle view, containing one to two plate-like plastids lying along the girdle (Fig. S1A). Valves were circular to elliptical with parallel striae (30 in 10 µm) and a distinct sternum along the long axis (Figs S1B, C). The striae consisted of rows of circular to elliptical areolae, which were occluded with complex vela and extended from the valve face onto the mantle (Figs S1C, D). The valve had marginal spines between the interstriae ( =  ribs, virgae) (Figs S1C, D). All these characteristics correspond to *Pseudostaurosira trainorii* as illustrated by Morales [17] and Morales et al. [18]. Mitotic cell division (not illustrated) was equal and followed immediately by the formation of new valves, as in other diatoms.

### Sexualization

Initially, gametogenesis was induced by mixing compatible clones. Later, experiments (see below) showed that sexualization could be induced by filtrates from cultures of the opposite sex. Sexualization was easily detected in living cells when, at the end of meiosis I, a cytokinesis occurred without the formation of new valves; this distinguished gametangia from mitotic cells, in which cytokinesis was always followed immediately by valve formation. The preceding meiotic prophase was difficult to detect in living cells because of the small size of the cell and nucleus. DAPI staining showed, however, that in both male and female clones, the nucleus enlarged considerably during the early stages of gametogenesis, as in all diatoms studied to date (e.g. [19]). After the cytokinesis at meiosis I, each daughter nucleus underwent an acytokinetic division at meiosis II and hence the maturing gametes were binucleate (Figs S2A–F). Fusion of gametes yielded zygotes with four nuclei (Fig. S2G), two of which subsequently degenerated, leaving two functional nuclei that remained unfused during auxospore expansion (Fig. S2H). The stages of sexual reproduction are dealt with in more detail below.

### Male gametogenesis

#### LM

**Figure 1:**
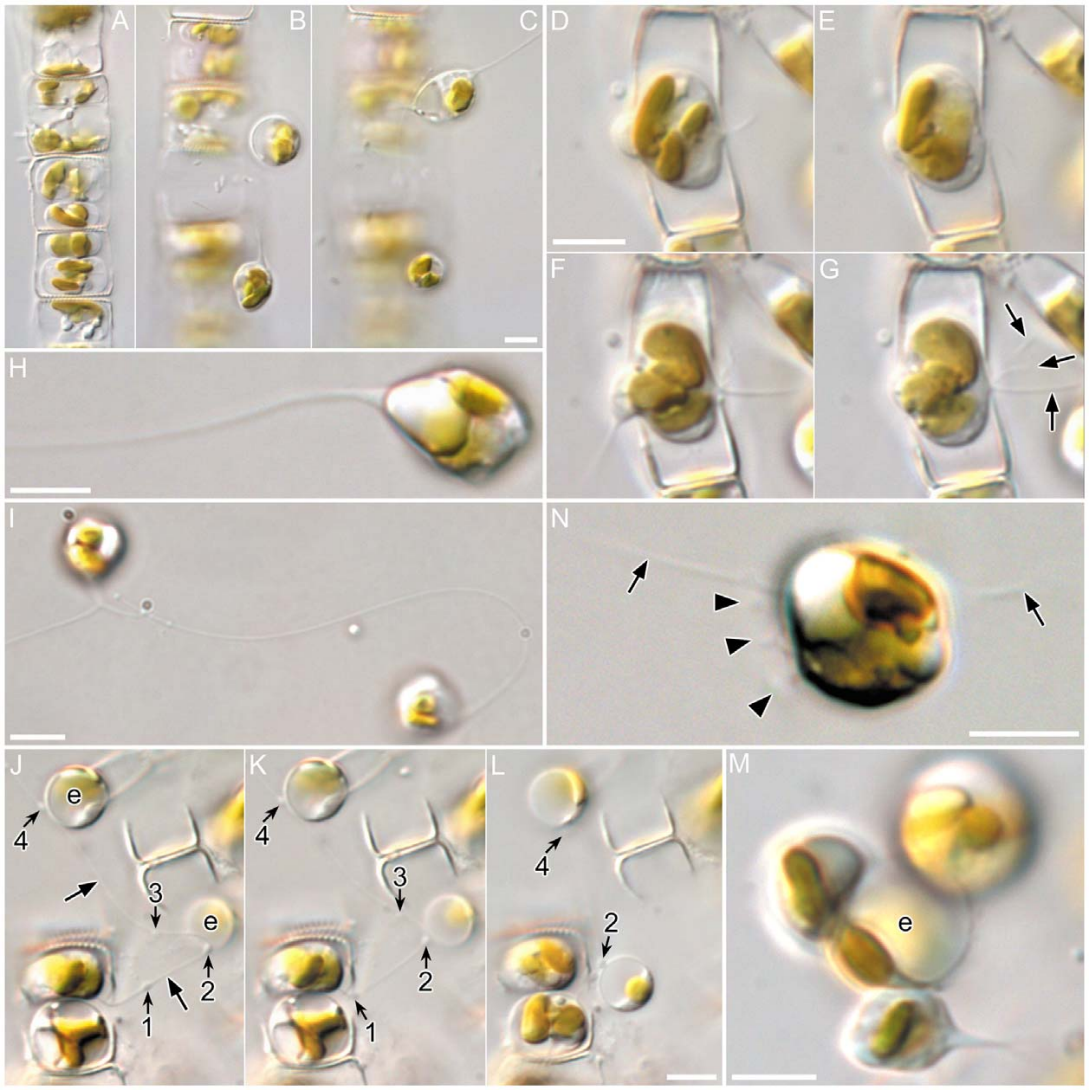
Male gamete formation and release in *Pseudostaurosira trainorii*. LM. Scales  =  5 µm. **A–C.** Sexualized male chain. **A.** Gametangia with two gametes. Note unequal size of gametes in a gametangium. **B–C.** Released gametes showing a thread extrusion in lower (B) and upper gamete (C). **D–G.** Male gamete actively moving within its gametangium and extruding threads from between the theca. Up to three threads can be seen (arrows in G). **H.** Gamete with a thread. The width of the proximal end of the thread widens slightly. **I.** Two gametes extruding the threads. Right cell has a long, highly bent thread. Two threads of the left cell are just fusing. **J–L.** Retrieval of egg with a condensate on the thread. **J.** Male gamete extrudes a thread from within its gametangium. Condensates are numbered. Two condensates, 2 and 4, have attached to egg cells (marked as e). **K.** Male gamete starts retrieving the thread. Note the positions of the condensates 1 and 2 have moved proximally. **L.** As the male gamete further retrieves the thread, one egg (bottom), which is attached by condensate 2, is drawn towards the male gamete. **M.** Three male gametes attach to an egg (marked as e). One male gamete (bottom) extrudes the thread. **N.** Male gametes with short projections. Arrow and arrowhead indicate thread and finer projections, respectively.

The cytokinesis accompanying the first meiotic division took place to one side of the median valvar plane of the gametangium, resulting in the formation of two gametes that were unequal in size (Figs 1A, J–L). The cytokinetic plane was mostly at the end of the hypotheca (so that the smaller gamete lay towards the epitheca: Fig. S3), but exceptions were observed (Fig. 1A) where possibly gametes had become rearranged. Maturing gametes swelled within the gametangia and pushed the gametangial thecae apart to become liberated (Fig. S3). Free gametes showed vigorous motility and many of them extruded fine threads (Figs 1B, C). Indeed, mature gametes often produced threads while still partially enclosed within the gametangium, the threads extending out into the medium from between the two gametangial thecae (Figs 1D–G, J–L). The threads were fine and transparent (Figs 1H, I). The direction of thread extrusion was apparently random, rather than aiming in a particular direction, and gametes often bore more than one thread (Figs 1F, G).

**Figure 2:**
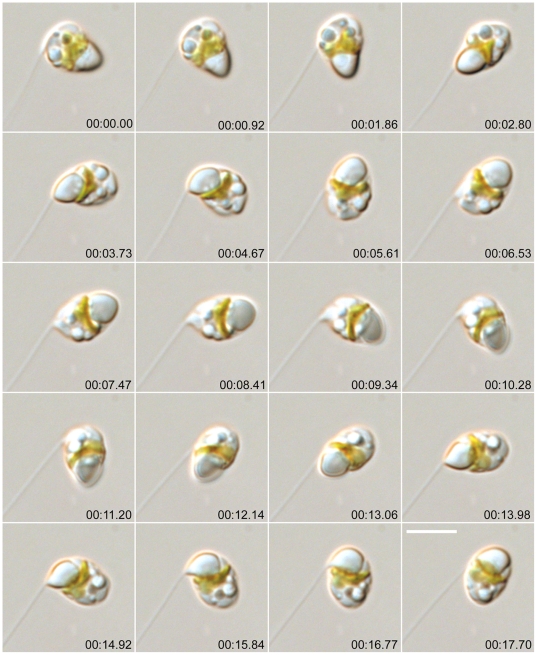
Male gamete in *Pseudostaurosira trainorii* retrieving the thread. Time lapse LM. Scale  =  5 µm. Cell spins at on its axis, remaining in the same place. The vacuole seems to be elastic as the shape is changed by the thread tension.

The gametic cells spun to reel in the threads (Figs 2, S4, Movie S1), often changing the form of the thread from straight to highly bent (Fig. 1I). The spinning accelerated as the gamete retrieved the thread and stopped when retrieval was complete (or sometimes before: Fig. S4). The threads exhibited no autonomous movement, except in a very few cases where the thread folded, apparently autonomously, as if there was a joint in it (Fig. S5, see also Fig. S8). The maximum length of the thread measured in this study was 84 µm, on a gamete whose diameter was 4.8 µm; such a thread could encircle the gamete equator c. 5.5 times when fully retrieved. No motion of the gamete was obvious while threads were being extruded.

Threads often developed expansions at points along their length. These expansions appeared ± homogeneous and similar in nature to the rest of the thread, rather than being extra material (e.g. bacteria, debris) adhering to the outside of the thread, or vacuolation of the thread. We refer to them here as ‘condensates’. As a gamete extruded a thread, the condensates were globular to slightly elongate and moved distally along with the thread, implying that the thread did not stretch or extend in the section where the condensates were located. When the gamete retrieved the thread, on the other hand, condensates became elongated as if stretched from both ends and finally became indistinguishable from the thread itself (Fig. S7). We frequently observed threads fusing from the base towards the tip to form one thicker thread (Fig. S6), whereas it was rare to observe threads separating, via a centripetal bifurcation. When two threads fused, a condensate was occasionally formed on the thread (Fig. S7).

Condensates seemed to be very sticky and they often attached to eggs if threads brushed against female cells (Fig. 1J). When such attached threads were wound by a male gamete, the attached egg was also retrieved to be fertilized (Figs 1K, L). In one case we observed a condensate on a thread attached to an egg that was still trapped within a gametangium; in this case the free male gamete was pulled towards the egg to fertilize it as the thread was retrieved.

**Figure 3:**
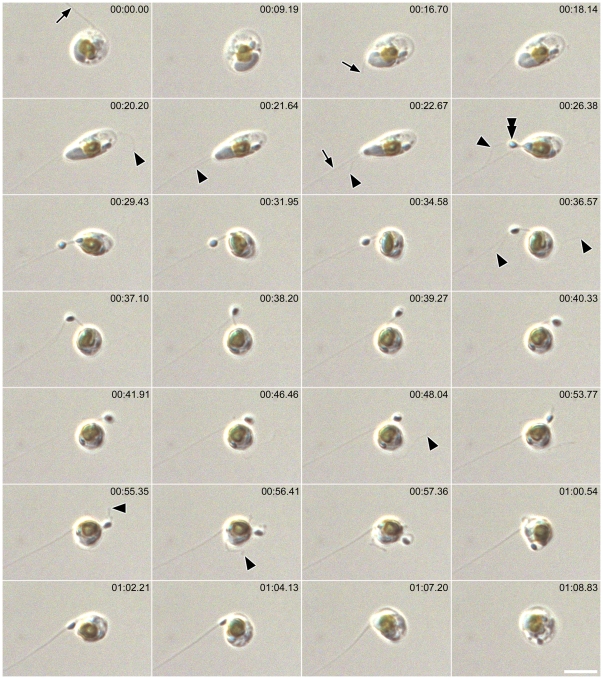
Male gamete in *Pseudostaurosira trainorii* with a blob. Time lapse LM. Scale  =  5 µm. The gamete retrieves the thread and then extrudes a blob with cell elongation. The blob is retrieved by the spinning gamete. Arrow and arrowhead indicate thread and finer projections, respectively. Double arrow indicates blob.

After thread retrieval, gametes often became highly elongated and secreted a blob from one end (Fig. 3, Movie S2). The blob and the gamete were linked by a short thread and the blob was swung around as the gamete spun. Further thread could be extended from the blob. Eventually the blob and the threads, both the thread linking to the blob and the thread extending from the blob, were retrieved together by the spinning gamete (Fig 3, Movie S2).

Threads could be produced throughout the independent lives of male gametes (i.e. before plasmogamy), including the last phases when they exhibited amoeboid movement on egg cells (Fig. 1M).

The gametes also extruded another type of projection, finer and shorter than the threads. These more delicate projections grew synchronously around the surface of the gamete, as well as at or near the proximal end of the thread (Figs 1N, 3, Fig. S8, Movie S2), and showed wobbling movements, typically lasting less than 10 seconds. Unlike the threads, the finer projections apparently moved autonomously (Movie S2).

#### SEM

**Figure 4:**
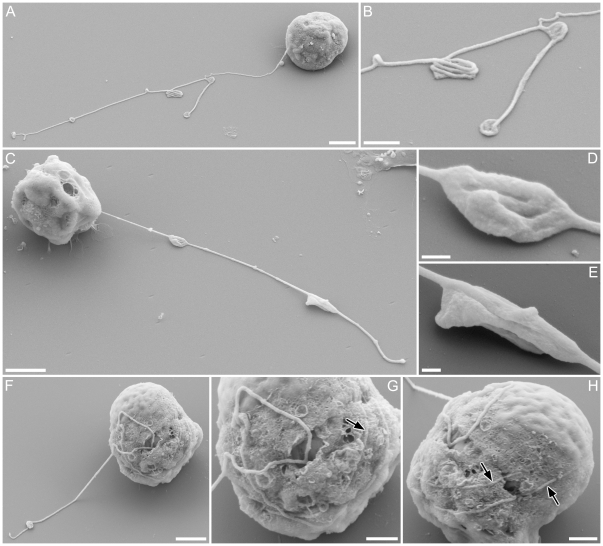
Surface fine structure of male gamete in *Pseudostaurosira trainorii* and its thread. SEM. Scales  =  2 µm (A, C, F, J), 1 µm (B, G, H) and 0.2 µm (D, E). **A.** Gamete with branched thread. **B.** Enlarged view of A showing condensates formed by folded threads. **C.** Gamete possibly retrieving a thread, judging from the elongated condensates, which are typically seen during thread retrieval. **D, E.** Enlarged view of C showing elongated condensates unfolding longitudinally (esp. E). **F.** Gamete winding the thread. **G.** Enlarged view of F showing a thread irregularly attached to the gamete surface. **H.** The same gamete as G observed from opposite side. Threads wound around the equator are visible.

**Figure 5:**
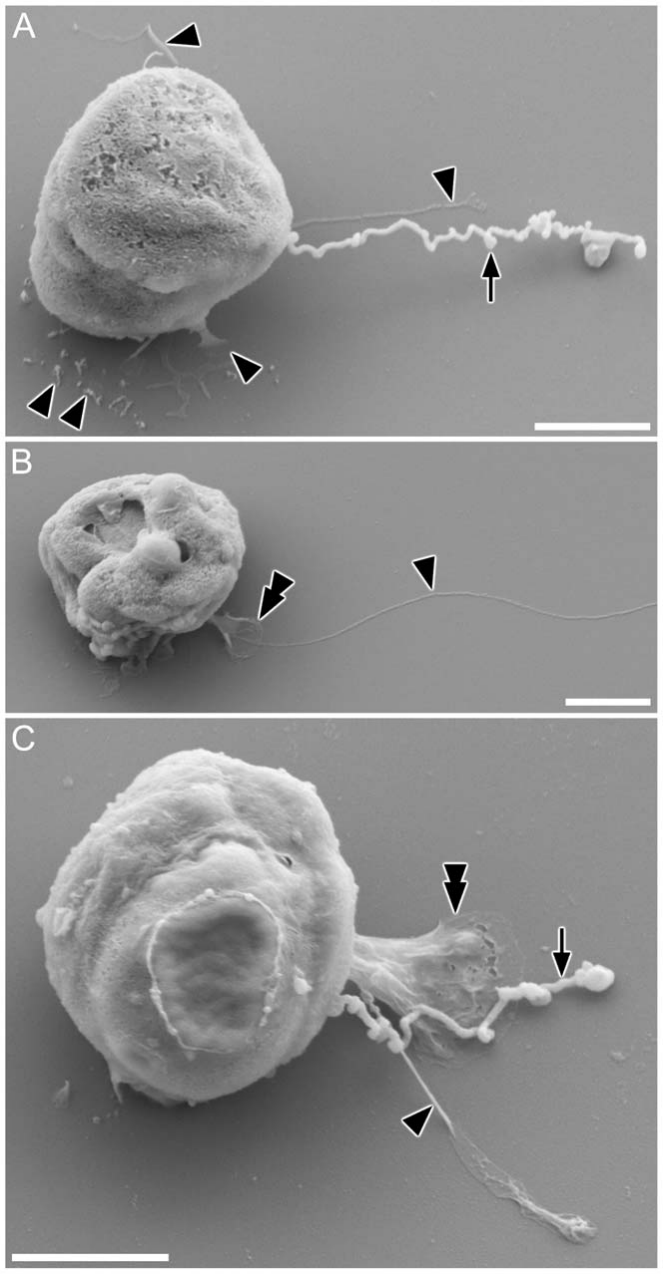
Surface fine structure of male gamete in *Pseudostaurosira trainorii* and its thread and projections. SEM. Scales  =  2 µm. **A.** Gamete with a thread as well as finer projections that vary in shape from a long type (top and middle, ± parallel to the thread) to a short, finger-like type (bottom). **B.** Gamete with a finer projection extending from a blob, corresponding to the LM observation in Fig. 3. **C.** Gamete with thread, blob and finer projection, the last of which seems to stem from the base of the blob. Arrow and arrowhead indicate thread and finer projections, respectively. Double arrow indicates blob.

Threads appeared plain, with no surface structure or mastigonemes (Figs 4A–J). The condensates on the thread appeared as densely folded or coiled threads, which were sometimes (Figs 4D, E) but not always (Fig. 4B, larger condensate) enclosed within a matrix (possibly the plasma-membrane). Along most of their length and between condensates, the threads appeared of almost uniform width (ca 10 nm), although LM observations indicate that threads often widen at their bases, where they emerge from the cell (Figs 1C, F, H). A gamete retrieving the thread around its equator was found, in which the thread seemed to partly be embedded inside the cell membrane (Figs 4F–H), rather than wound outside the cell. The finer projections noted in LM were distinguishable from threads by their thickness (Figs 5A–C; arrowheads). Collapsed and flattened material apparently corresponding to the blobs observed in LM were visible adhering to the stub after critical point drying, with well-preserved membrane structure (Figs 5B, C; double arrowheads).

### Confocal fluorescence microscopy

**Figure 6:**
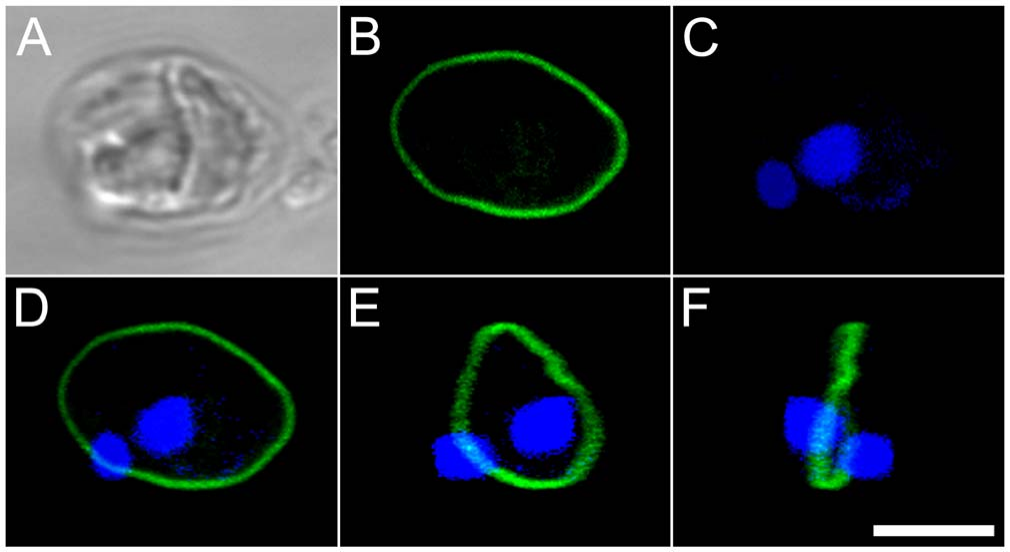
Male gamete in *Pseudostaurosira trainorii* with no extruded thread. LM. Scale  =  5 µm. **A.** Bright field. **B.** Tubulin immunolocalization. **C.** DNA staining with DAPI. **D.** Merged image of B and C. **E, F.** Three dimensional reconstruction based on 38 optical stacks taken every 0.08 µm and rotated ca. 45° (E) and 90° (F). Note that tubulin is localized on the equator of the gamete to form a microtubule ring.

**Figure 7:**
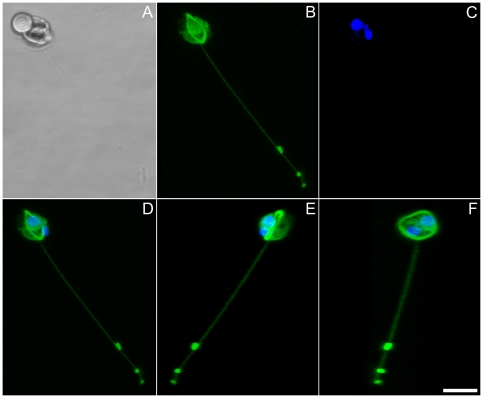
Male gamete in *Pseudostaurosira trainorii* extruding a thread. LM. Scale  =  5 µm. **A.** Bright field. **B.** Tubulin immunolocalization. **C.** DNA staining with DAPI. **D.** Merged image of B and C. **E, F.** Three dimensional reconstruction based on 33 optical stacks taken every 0.21 µm and rotated ca. 130° (E) and 80° (F). Note that a thread stems from the microtubular ring.

A clear anti-tubulin signal was detected as a ring on the equator of globular gametes (Fig. 6) and along the length of extruded threads (Figs 7, S9). Condensates were particularly strongly stained, confirming that these are masses of densely folded thread material. Three dimensional analyses revealed that the thread stemmed from the equator ring (Figs 6E, F, 7D, E). The finer projections were also labelled with antibody, although their exact origin could not be resolved.

### Female gametogenesis

**Figure 8:**
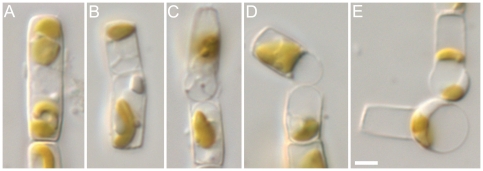
Female gamete formation in *Pseudostaurosira trainorii*. LM. Scale  =  5 µm. **A.** Plastids appressed to valve at the beginning of miosis. **B.** Cytokinesis takes place not at the middle of the gametangium but offset. **C.** Divided surfaces of gametes expand. **D.** Each gamete becomes rounded on the side facing its sibling and pushes against it, causing separation of the thecae. **E.** Mature gamete.

Gamete formation in female clones was almost the same as in males. The plastids were appressed to the valve prior to the meiosis I cytokinesis (Figs 8A, B), which was slightly offset from the cell equator to create two unequal gametes. As in males, the gametes rounded and swelled to open the gametangial frustule (Fig. 8C), but then each gamete swelled further (Fig. 8D) and moved to the open end of the theca in which it had been formed, settling there as a spherical egg cell (Fig. 8E). Mature eggs were highly vacuolate and contained one or two plastids and a nucleus, all situated peripherally (Fig. 8E). No threads or projections were formed by egg cells.

### Fertilization

**Figure 9:**
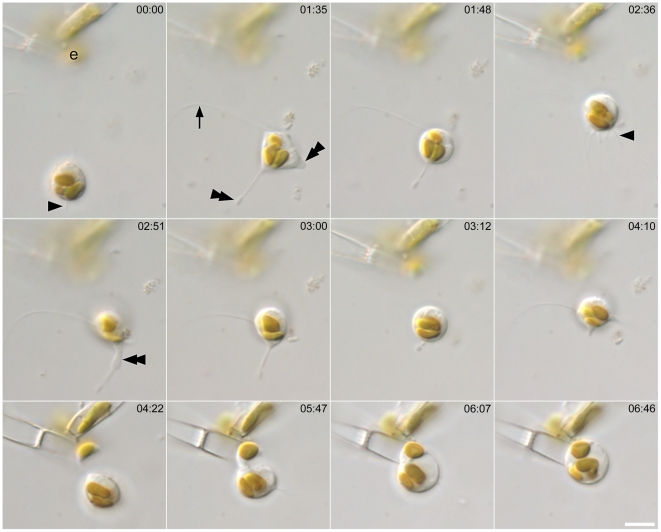
Approach of male gamete to egg and fertilization in *Pseudostaurosira trainorii*. Time lapse LM. Scale  =  5 µm. Male gamete moves towards the egg (marked as e), extruding a thread (arrow), finer projections (arrowheads) and pseudopodium-like structure (Double arrow).

The initial movements of male gametes, generated by thread formation and retrieval, were non-directional, comprising a ‘random walk’. However, when a male gamete came within close range of an egg after random walk, it headed directly towards the egg, changing from globular to a less definite shape and becoming amoeboid (Figs 9, S10). Pseudopodium-like extensions were visible (Fig. 9; double arrowheads), which changed their shape dynamically and were consistently thicker than the threads and finer projections, which continued to be extruded actively (Fig. 9; arrow and arrowheads, respectively).

Male cells were sometimes larger, sometimes smaller than the eggs they fertilized, depending on the relative sizes of the vegetative cells (and hence of the gametangia) in the two clones mated. For example, Fig. 9 shows fertilization of a small female by a larger male.

Plasmogamy took place within a few seconds or minutes after the contact of compatible gametes. Exceptionally (< 10% of copulations), eggs were unable to fuse with males despite the movement of male gamete over its surface for more than 10 min, apparently trying to penetrate its membrane. The male gametes were seemingly healthy, judging by their success in reaching the eggs and their active movement over the egg surface. Once male gametes attached to an egg, they never left it. Just after plasmogamy, the zygote absorbed any threads derived from the male gamete, without any spinning motion.

Male gametes were attracted out of their random walk not only by exposed egg cells but also by immature female gametangia whose gametes were as yet unexpanded within a closed frustule. In this case, male gametes often positioned themselves around the junction between the female thecae, waiting for the eggs to mature. They fertilized them as soon as the female gametangia opened (Fig. S11).

In one case, we observed an amoeboid male gamete moving towards an egg that was being fertilized by another male gamete (Fig. S12). When fertilization occurred, the moving gamete stopped its approach and then moved around the zygote, apparently at random.

**Figure 10:**
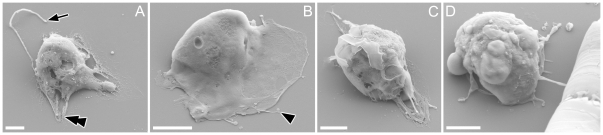
Male gamete in *Pseudostaurosira trainorii,* showing the morphology in the amoeboid phase. SEM. Scales  =  2 µm. **A. A t**hread is extruded from the pseudopodium-like structure. **B.** Short projections are visible on the edge of the pseudopodium-like structure. **C.** Highly elongated cell extending pseudopodia at both ends. **D.** Male gamete attaching to a female gametangium by its thread. Arrow and arrowhead indicate thread and finer projections, respectively.

SEM observations of male gametes in the amoeboid stage showed the presence of broad or narrow, sheet-like pseudopodia, from which threads and finer projections were often extruded (Figs 10A–D).

India Ink preparations of gametes and zygotes revealed no mucilage envelope around the gametes (Figs S13A, B) and young zygotes (Fig. S13C). However, a globular envelope (likely mucilaginous) surrounded older, spherical zygotes (Figs S13A–F), separating them from the gametangial thecae to which they were originally attached. The mucilage envelope remained spherical as the zygote began to expand to form highly elongated auxospore (Figs S13E, F). SEM observations confirmed the absence of the mucilage envelope on male gametes (e.g. Fig. 4) and eggs (Fig. S13F) and its presence on zygotes (Figs S13H, I).

### Pheromone experiments

Experimental design, brief results, and their implications are summarized in Table 1:

- Expt. 1. Successful fertilization took place in both continuous light and continuous dark. There was no obvious difference in the amount of sexualization between the two treatments.
- Expt. 2. Gametes were released in both clones (Figs S14A, B) when these were placed in different holes separated by solid agar (Fig. S15A). Male gametes extruded and retrieved threads with spinning motion, but unlike Expt. 1 and other successful crosses, no amoeboid movement was seen.
- Expt. 3. Male clones mixed with filtrates of female vegetative clones became sexualized and released gametes, which remained solitary and died since eggs were absent.
- Expt. 4. No sexualization was seen in female clones supplied with filtrate from male vegetative cells. Hence females initiate sexual reproduction but they do not become sexualized in the absence of males.
- Expt. 5. Female clones become sexualized mixed with filtrate from sexualized male clones comprised predominantly of released gametes and immature gametes still enclosed within gametangia, but also some remaining vegetative cells.
- Expt. 6. Amoeboid movements and the attraction of motile male cells to females was not triggered if the male gametes were mixed with vegetative females, as opposed to sexualized clones containing immature or mature female gametangia (Fig. S14C).
- Expt. 7. Male clones became sexualized when incubated with agar blocks containing filtrates of either vegetative or sexualized female cells (Fig. S15B). As in Expt. 5, it was not necessary for the females to be in the final stages of egg production to induce sexuality in males. *In situ* observations showed that released male gametes remained near the gametangia that produced them, showing extrusion and retrieval of the threads with spinning motions but without exhibiting amoeboid movement (Fig. S14D).
- Expt. 8. Both male and female clones became sexualized and released gametes when grown together within the same culture dish but prevented from contacting each other by incubation in separate compartments (Fig. S15C), indicating that diffusible signals passed between them. The male gametes remained in the vicinity of the gametangia producing them after liberation, with no net movement towards the females (which were c. 1 mm distant).
- Expt. 9. Although some male gametes happened to come in contact with dead eggs as a result of their random walk, they left the eggs after a few minutes (Fig. S14E). Directional amoeboid movement toward the eggs was not seen. To confirm that females had been killed by the heat or UV treatments, a female clone was treated in the same way and put in fresh medium: no growth occurred.

**Table 1:** Summary of pheromone experiments in *Pseudostaurosira trainorii*.

Experiment	Treatment/conditions	Results	Indications
1: ♂×♀	(1) 24h light; (2) 24h dark	successful fertilization	*Pseudostaurosira trainorii* is day-neutral
2: ♂×♀	each clone placed in a separate hole in an agar plate (Fig. S15A)	both clones sexualized. No amoeboid movement of ♂gametes.	compatible clones can interact through solid agar, most likely by means of chemical signals, which however do not include the one that triggers amoeboid movement of male gametes
3: ♂×F[♀]	in liquid medium	♂ sexualized (Fig. S14A)	vegetative ♀ secrete sex pheromone (ph-1)
4: ♀×F[♂]	in liquid medium	♀ not sexualized	vegetative ♂ secrete no sex pheromone
5: ♀×F[♂S]	in liquid medium	♀ sexualized (Fig. S14B).	sexualized ♂ secretes sex pheromone (ph-2)
6: ♂S×♀	in liquid medium	no movement of ♂ gametes towards vegetative ♀ (Fig. S14C).	ph-1 does not trigger the amoeboid movement of ♂ gametes. A putative sex pheromone (ph-3), which is the attractant for the ♂ gametes, can only be secreted from sexualized cells
7: ♂×F[♀] and ♂×F[♀S]	in gel medium (Fig. S15B)	♂ sexualized in both gels, but no amoeboid movement of ♂ gametes (Fig. S14D)	active ph-1, presumably secreted from both ♀ and ♀S, can be enclosed in gel, while ph3, if any , cannot be retained in gel.
8: ♂×♀	♂ and ♀ separated physically in different compartments connected by a bridge of culture medium (Fig. S15C)	both clones sexualized, but no amoeboid movement of ♂ gametes	the effective range of ph-3 is smaller than for ph-1 and ph-2
9: ♂S×dead ♀S*	in liquid medium	no amoeboid movement of ♂ gametes (Fig. S14E)	physical contact between threads and egg cells is unlikely to be required to trigger amoeboid movement of ♂ gametes

All experiments were performed in triplicate and the results were consistent. Unless stated otherwise, ♂ and ♀ are vegetative cells of male and female clones, respectively; S  =  sexualized clone where released gametes and gametangia were predominant with a small amount of vegetative cells; F  =  filtrate, so that F[♀}, F[♂S], etc. are filtrates of females, male sexualized clones, respectively.

*♀ gametes were killed by heating to 98°C for 1 h, or leaving Eppendorf tubes under 321 nm UV light (with TFX-20M transilluminator: Life Technologies, Inc, Paisley, UK) for 1 h.

## Discussion

### Thread and the other projections

The male gametes of *Pseudostaurosira trainorii* possess structures, which we term ‘threads’, that are hitherto unknown in diatoms and other stramenopiles. The threads of *P. trainorii* are not flagella and differ from them (including the flagellum of sperm in centric diatoms) in many ways, both functionally and morphologically. In *P. trainorii* the gamete controls the movement of the thread, which cannot itself beat or undulate and has no motility of its own. Away from the cell body, threads are generally straight and appear to be stiff, since they flex into even, gentle curves during retrieval, if the end of the thread is attached. On the other hand, they can bend abruptly, as if jointed (Figs S5, S8), and can coil tightly, as for example in the ‘condensates’. Threads are sticky and catch and draw objects, including eggs, towards or away from the male gamete, depending on whether the thread is being retrieved or extended; the condensates appear to be particularly sticky and thus increase the chance of attachment. The motility of the male gametes conferred by the threads is probably crucial for sexual reproduction of *P. trainorii*, because this diatom, as a member of the araphid pennate group, is non-motile in its vegetative stage but obligately outbreeding. In centric diatoms, the same purpose is served by the flagellum of the sperm, which create motility by their autonomous beating, which leads the sperm directly to the egg (e.g. [20]).

Our confocal results show unambiguously that the threads contain microtubules, which become wound tightly around the cell body during retrieval, rather than being depolymerized from the base (depolymerization from the tip is ruled out by the behaviour of the condensates, which maintain a constant distant from the thread tip during both retrieval and extension). The possession of a microtubular skeleton clearly differentiates the threads from the filopodia of rhizarians such as foraminifera and filose testate amoebae (e.g. *Euglypha*) [21] and links them more closely to the axopodia of heliozoan amoebae, some of which, e.g. *Raphidiophrys*, are thought to be related to the stramenopiles within the chromalveolate–rhizarian group [22]. However, among the projections and pseudopodia produced by different protists, the most similar to the threads in terms of size and behaviour is probably the haptonema of Haptophyta. Like the threads, haptonemata bend and coil (forming tight coils, as in the condensates) but do not beat, and they are also sticky, being used for attachment (e.g. see [23], [24]) and also for ‘fishing’, in this case for prey during mixotrophy rather than for a sexual partner [25]. Any similarities between the threads and haptonemata are likely to reflect convergent evolution, since *Pseudostaurosira* and haptophyte algae are only very distantly related (e.g. [22]) and intervening lineages of protists (e.g. centric diatom lineages, bolidophytes, other stramenopiles) lack haptonema-like structures. Nevertheless, it is worth exploring whether similar behaviour (e.g. tight coiling) may be based on similar mechanisms, e.g. control via calcium fluxes (cf. [24]).

The functions of the blob and finer projections in the male gates are unclear. After critical point drying, blobs collapsed and seemed to be left empty. The interior of the blob may therefore be filled with a watery or oily material, rather than mucilaginous substances like those forming the capsule secreted around the zygote, which retains its integrity, and to a lesser extent its shape and volume, after critical point drying (Fig. S13H).

### Male gamete motility

Male gametes exhibit several types of activity: 1) thread extrusion, with no spinning or amoeboid movement, 2) spinning motion during thread retrieval, 3) extrusion of finer projections from settled cells, 4) extrusion of a blob with accompanying cell elongation and 5) amoeboid movement, which, so far, has been seen to occur only when compatible clones are mixed. Significant movement of the whole cell (by more than a cell length) can occur through activities (1) or (2) when the thread attaches to a substratum. Cells also move with (5) but this is less easily detected under low magnification because the speed is relatively low and the cells do not spin when they move, unlike (1) or (2). Some types of activity can be concurrent, e.g. a cell extruding a thread can also extrude finer projections or a blob, and amoeboid gametes can extrude threads. The gamete behaviour most commonly observed is repeated extrusion and retrieval of threads.

Male gametes move vigorously by extruding and retrieving threads. When a part of a thread, most likely a sticky condensate, happens to attach to a large object or other cells, the gamete is drawn to the attached part as it spins to retrieve the thread. If the thread happens to attach to an egg, the male gamete can fertilize the egg either by retrieving the egg with the thread, or by drawing itself towards the egg during retrieval, if the female gametangium is fixed to the substratum.

We observed many male gametes that did not find egg cells even when the eggs were within the reach of the threads; in some cases the threads made a ‘near miss’ but sometimes they extended in completely the wrong direction. Therefore, it is likely that the male gametes extrude threads randomly, rather than aiming them in the direction of an egg and their success in catching an egg with this strategy depends totally on chance.

The more important mechanism for promoting fertilization seems to be amoeboid movement. Although the male gametes may need to be close to the egg to detect the signals that trigger the amoeboid phase, the amoeboid male gamete will then certainly find egg cells through directional movement.

Comparisons with other pennate diatoms are hindered by the lack of information on most other araphid pennates. The mode of sexual reproduction in pennate diatoms is highly diverse [13], [26] with respect to whether or not cells pair actively (raphid diatoms do, araphid pennates apparently do not) and the ways in which cells copulate and produce gametes [27]. The araphid pennates that have been studied thus far generally mate via distant pairing, where the gametangia are not in contact at any time during gametogenesis, as in *Pseudostaurosira*. However, these diatoms appear to lack threads and compatible cells must be brought together passively and grow close together for plasmogamy to occur, due to their immobile nature of the vegetative cells and the limited motility of their gametes. In the araphid *Licmophora*, for instance, Chepurnov and Mann [14] observed that in successful pairs, gametangia were mostly close enough to touch, but that in a few cases plasmogamy could occur when gametangia were roughly a cell’s length apart. Although motility of vegetative cells is known in *Licmophora* [28], the cells studied by Chepurnov and Mann [14] were attached to the substratum by mucilaginous stalks. In the anisogamous araphids *Rhabdonema* [15] and *Grammatophora* [16], fragmented chains of males gametangia become attached to female chains and there release their gametes, which are able to move a short distance by amoeboid movement. In all of these cases, the motility of the gametes is not as dynamic as in *P. trainorii.*

### Pheromones

**Figure 11:**
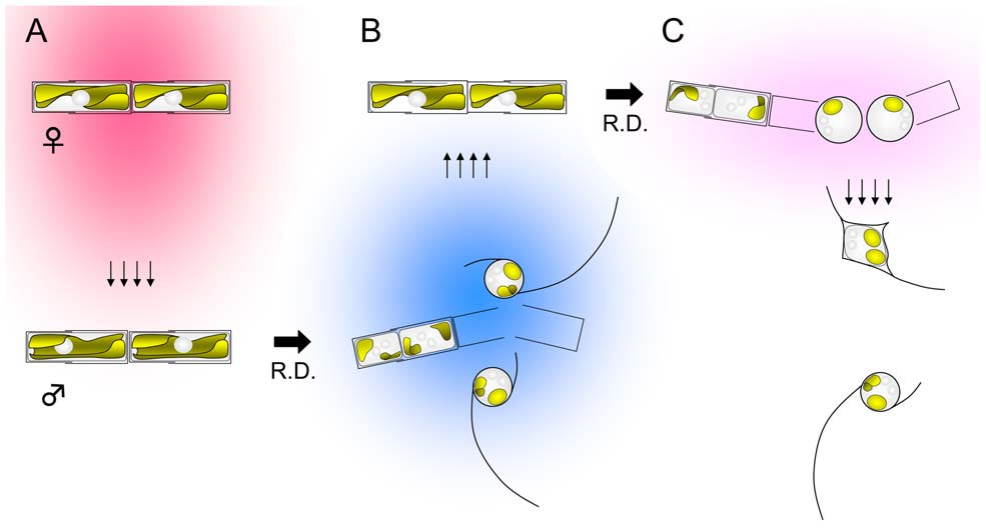
Schematic illustrations to summarize the process of pheromonal interactions during sexual reproduction in *Pseudostaurosira trainorii.* **A.** Vegetative female cells secrete ph-1 (shown in pink) which stimulates sexualization of male cells. **B.** Sexualized male cells secrete ph-2 (shown in blue) which stimulates sexualization of female cells. **C.** Sexualized female cells secrete a putative pheromone ph-3 (shown in light pink) which directs the male gametes and triggers their amoeboid movement.

In our study the existence of two sex pheromones has been demonstrated by experiment. The process of sexualization is as follows: sexualization of male clones is triggered by a female sex pheromone ph-1 which seems to be secreted constitutively by female vegetative clones in the sexual size range when growing actively in our culture conditions (Fig. 11A). Given the presence of ph-1, male vegetative cells undergo meiotic divisions and release two motile gametes. The sexualized male cells and/or gametes secrete ph-2, which stimulates the sexualization of female cells (Fig. 11B).

To our knowledge, strategies similar to that of *P. trainorii* using ph-1 and -2 have not been found in brown algae, which are close relatives of diatoms and whose sex pheromones have been intensively investigated (e.g. [29], and refs therein). Nevertheless, chemical signalling is clearly important during brown algal reproduction. Chemokinesis (phobo-chemotaxis) is seen in *Fucus spiralis* and *Hormosira banksii*, in that spermatozoids exhibit characteristic U-turns when heading away from a pheromone source [30], [31]. On the other hand, *Laminaria* sperm seem to exhibit chemotaxis (topo-chemotaxis), i.e. spermatozoids show a directed movement towards a pheromone-secreting egg [29]. In *Laminaria*, and also in *Ectocarpus siliculosus*, spermatozoids tend to show thigmotactic swimming (i.e. in contact with the surface of a substratum), a phenomenon which is strongly enhanced by the presence of pheromone [29], [32]. The behaviour of *P. trainorii* male gametes when they head towards eggs resembles *Laminaria*, i.e. males are directed towards eggs via amoeboid movement (a form of thigmotaxis): however, ph-1 secreted from female cells of *P. trainorii* does not appear to stimulate the directed movement of male gametes. Thus, it is reasonable to assume that the egg has a mechanism to attract male gametes that was undetected in this experiments.

Another stramenopile alga, the chrysophyte *Dinobryon cylindricum*, may secrete sex pheromones from female vegetative cells [33], but reciprocal stimulation is unknown. Among the oomycetes, which are a group of non-photosynthetic stramenopiles that appears to be basal to the diatoms and other autotrophic stramenopiles in molecular phylogenies (e.g. [34]), pheromonal interactions very similar to those of *Pseudostaurosira* have long been known in *Achlya* [35]. Female thalli produce antheridiol, which induces differentiation of antheridial branches. Male thalli, in turn, produce oogoniols, which initiate the formation of oogonia (summarized by [36], [37]), but, as with *Pseudostaurosira* ph-2, oogoniols are not produced by vegetative male thalli, only by sexualized antheridial branches. An interaction between compatible clones like that involving ph-1 and -2, is also known in *Closterium* [38] but such green algae are only very distantly related to the stramenopiles.

Amoeboid movement of male gametes was observed when compatible clones were mixed, but not when males were exposed to filtrates of vegetative or sexualized females nor when females or filtrates were contained within an agar gel. Thus, the triggers for amoeboid movement could be (1) physical contact between the thread and the egg, (2) high concentrations of ph-1, that were not replicated in our experiments, or (3) an additional pheromone, ph-3, which is secreted from sexualized female cells but cannot be retained in filtrates or agar gels.

The first possibility is made less likely by the results of Expt. 9, where no amoeboid movement of male gametes was seen when they were mixed with dead eggs treated with heat shock or UV exposure. However, it is possible that key receptors were damaged, even though alteration of surface proteins should have been minimal with UV treatment (heat treatment can be expected to be more drastic).

Expt. 6 appears to makes the second possibility (high concentrations of ph-1 induce amoeboid movement) unlikely. Male gametes sometimes attached to vegetative female cells, but this was probably because the threads happened to attach to female cells and brought the male gametes and female cells together when the gametes span to retrieve the threads. In this experiment female cells eventually attracted the male gametes, but it took place several hours after two clones were mixed, so that the females may have been sexualized. On the other hand, by analogy with *Achlya* (in which antheridiol serves both for stimulation of antheridium development and attraction of antheridial hyphae), it may be that the females of *P. trainorii* are induced to produce more ph-1 when stimulated by ph-2, against a background of lower, constitutive production from vegetative cells.

Overall, however, we consider the ph-3 hypothesis (Fig. 11C) to be the most reasonable explanation of the present observations. Male gametes left dead eggs after a few minutes (Fig. S14E), suggesting that chemical signalling from a living cell is important in the initial recognition between compatible gametes. Ph-3 may be very ephemeral or highly volatile so that it is not retained by agar gels (Expt. 7) and so that the male gametes received no signal in Expt. 8.

Male gametes do not leave a living egg once they have attached to it. We sometimes observed an egg with more than one male gamete (Fig. 1M), suggesting that the eggs keep attracting male gametes even when one has already attached. However, fusion of several male gametes with a single egg was not observed, suggesting effective polyspermy blocks (cf. brown algae [39]). Secretion of ph-3 seems to stop soon after fertilization, judging from the observation shown in Fig. S12, where the fused egg seemed to lose a signal that attracted the male gamete as the amoeboid male gamete then wandered around the zygote.

Pre-ectocarpene, a sex pheromone extracted from *E. siliculosus*, is transformed by a Cope-rearrangement into the inactive ectocarpene [40]: this is thought to be a mechanism of fast signal inactivation by spontaneous intramolecular rearrangement [29]. It would not be surprising if the same or similar mechanism had been adopted in diatoms, since they are close relatives of the brown algae and some of them are known to produce compounds that were originally found from brown algae, e.g. octadiene and fucoserratene (the *Fucus* pheromone) in *Asterionella formosa* [41], and ectocarpene in *Skeletonema costatum* [42], *Asterionella* and *Gomphonema* [29], though none of these compounds have been proven to be sex pheromones in diatoms.

### Induction of sexuality

In all the diatoms, sexuality is size-dependent and sexual reproduction can only be induced below a threshold [43], [44]. When the cells enter the sexual size range, the induction of the sexual reproduction in centrics, which are all homothallic, is solely influenced by environmental factors (e.g. light intensity, photoperiod, temperature or salinity). On the other hand, in the pennates (mostly allogamous), cell–cell interactions between compatible cells may be the primary determinant of when and where sexual reproduction occurs (e.g. [14], [45] for araphids, [46] for raphid, [47], [48] for reviews), although external factors are also important. Thus, *P. trainorii* is the first pennate shown to be capable of being sexualized in the absence of compatible cells, i.e. sexuality can be induced by filtrates of the opposite sex. It is also worthwhile to note that no external factor limits the induction of the sexuality in *P. trainorii*: sexualization occurred under any light conditions (photoperiod and intensity) or temperature tested so far (Sato unpubl.).

## Materials and Methods

### Ethics statement

No specific permits were required for the sampling as the location is not privately-owned or protected in any way, and the field studies did not involve endangered or protected species.

### Collections, cultures

Vegetative cells of the *Pseudostaurosira trainorii* were collected from bottom sands at Obuchi-numa Lake, Aomori Pref., Japan on 25 July 2010. Single cells were isolated from the sample to obtain clonal cultures. Among 18 clones successfully established (6 male and 12 female), clone number 3 (male) and 7 (female) were used for all the observations and experiments. Cultures were maintained in a 1∶1 mixture of WC medium + silicate [49] and Roshchin medium [50] at 15°C under cool-white fluorescent light on a 14∶10 (L:D) photoperiod at a photon flux density of 5–20 µmol photons m^–2^ s^–1^.

### Induction of sexuality

Gamete release was induced using agar plates as in pheromone experiment Expt. 2 (Fig. S13A), where compatible clones were put into separate holes physically separated (by 3% solid agar and ca. 1 cm apart. Since this agar method enabled us to collect populations of solely male or female gametes, most of the microscopical observations were conducted using these specimens, rather than mixtures of compatible clones. The exceptions were for observations of fertilization, where two compatible clones were mixed in liquid medium prepared as above.

### Light microscopy

Living cells were observed with an Axio Imager M2 with Axiocam MRc5 digital camera (Zeiss, Oberokochen, Germany) and differential interference contrast (DIC) optics. The objective used was a 100× planapo, NA 1.4. Time-lapse images were also obtained with this LM, using Zeiss Axiovision software.

### Fluorescence observation

For epifluorescence microscopy, male gametes were fixed with 2.5% glutaraldehyde at room temperature for 1 h and rinsed three times with PBS buffer containing 1.5 % NaCl. Cells were stained with DAPI at a concentration of 1 µg ml^–1^ and observed with a Zeiss AxioPhot 1 microscope with an AxioCam MRm camera.

Microtubules were stained using antibodies. Cells were fixed with 4% paraformaldehyde in PBS at room temperature for 1 h, then rinsed three times with the same buffer and incubated 1 h in PBS containing 0.1% Triton X-100. Cells were then rinsed once and incubated 30 min in PBS containing 1% BSA. Anti alpha-tubulin conjugated with Alexa Fluor 488 (eBioscience Ltd, Hatfield, UK) was used at a concentration of 5 µg ml^–1^ at room temperature for 2 h. DAPI was also added in this process at the same concentration as for epifluorescence observations. Cells were rinsed three times and mounted with Fluoromount/Plus (Diagnostic BioSystems, USA). A SP5 Confocal Microscope (Leica Microsystems, Heidelberg, Germany) was used for observation. Three dimensional modelling was reconstructed with confocal stacks using Image J software [51].

### Electron microscopy

For SEM examination, male gametes were mounted on poly-L-lysine coated cover slips, or compatible clones were crossed on these slips, and fixed with 2.5 % glutaraldehyde for 1 h at room temperature. Cover slips were rinsed with PBS three times and post-fixed with 1% osmium tetroxide for 1 h at room temperature. The specimens were dehydrated through a graded series of ethanol and then infiltrated with acetone and dried with a Emitech K850 critical point dryer (EM Technologies, Kent, UK). The cover-slips were then attached to aluminium stubs, coated with platinum for 1 min in an Emitech K575X sputter coater, and examined using a LEO Supra 55VP Field Emission SEM (LEO Electron Microscopy, Oberkochen, Germany) operated at 5 kV (5 mm working distance; aperture 20 µm).

### Pheromone experiments

Compatible clones were crossed in order to examine the properties of sex pheromones. All experiments were in triplicate and conducted at 15°C, using 50 mm Petri dishes, 0.2 µm nylon filters (Millipore Corp., Bedford, MA, USA) to prepare filtrate, and agar (3%). Details of experiments are summarized in Table 1.

## Supporting Information

Figure S1 **Vegetative cell in** ***Pseudostaurosira trainorii.*** LM (A, B) and SEM (C, D). Scales  =  5 µm (A), 2 µm (B, C) or 0.5 µm (D). **A.** Living cells attaching to each other to form a ribbon colony. **B.** Acid-cleaned valve showing parallel striae. **C.** Striae consisting of single rows of areolae. Valve with marginal spines. **D.** Enlargement of areolae, which are occluded by complex vela.
(TIF)

Figure S2 **Nuclear behaviour in** ***Pseudostaurosira trainorii*** **visualized with DAPI.** LM. Scale  =  5 µm. A–C. Male clones. D–F. Female clones. G, H. Zygote. Bright field (1) and fluorescence image (2). **A.** Large nucleus at the centre, plastids appressed to valve. **B.** One nucleus per gamete after meiosis I. **C.** Meiosis II results in two nuclei per gamete. **D.** A large nucleus at the centre. **E.** Meiosis I results in two nuclei in a gametangium. **F.** Further nuclear division at meiosis II. Cytokinesis does not occur at meiosis II, resulting in four nuclei per gametangium, two in each gamete. **G, H.** Zygote observed under different focuses. The early stage shown in G lacks a mucilage envelope and contains four nuclei, two paler than the others; at a slightly later stage (H), the zygote is covered by a mucilage envelope and contains only two nuclei.
(TIF)

Figure S3 **Male gamete liberation in** ***Pseudostaurosira trainorii*** **.** Time lapse LM. Scale  =  5 µm. The two gametes are unequal in size. The larger gamete (right) swells out from the gametangium.
(TIF)

Figure S4 **Retrieval and extrusion of thread of male gamete in** ***Pseudostaurosira trainorii*** **.** Time lapse LM. Scale  =  10 µm. A long thread is retrieved as the gamete spins, and then extruded again unless the thread is fully wound.
(TIF)

Figure S5 **Autonomously folded thread on male gamete in** ***Pseudostaurosira trainorii*** **.** Time lapse LM. Scale  =  5 µm. Note the thread is folded near to the root from 00:04.58 onwards.
(TIF)

Figure S6 **Fusing threads on male gamete in** ***Pseudostaurosira trainorii*** **.** Time lapse LM. Scale  =  5 µm. Gamete spins to retrieve threads (until 00:15.88), which are then fused from proximal end (00:16.51 onwards). Note the gamete does not spin when the threads are fusing.
(TIF)

Figure S7 **Thread behaviour of male gamete in** ***Pseudostaurosira trainorii.*** Time lapse LM. Scale  =  5 µm. When two threads fuse, a condensate (arrowhead) is formed at the bottom of the threads. The condensate moves distally as the gamete extrudes the thread, and then it moves back again proximally as the gamete retrieves the thread. Note that the shape of the condensate is somewhat globular when the thread is extruded, whereas it elongates and disappears at the end when the thread is retrieved.
(TIF)

Figure S8 **Finer projections of male gamete in** ***Pseudostaurosira trainorii.*** Time lapse LM. Scale  =  5 µm. Finer projections are seen on the gametic surface as well as on the thread. Arrow and arrowhead indicate thread and finer projections, respectively.
(TIF)

Figure S9 **Male gamete extruding a thread. LM. Scale  =  5** µ**m.** **A.** Bright field optics. **B.** Tubulin immunolocalization. **C.** DNA staining with DAPI. **D.** Merged image of B and C. **E, F.** Three-dimensional reconstruction based on 27 optical stacks taken every 0.21 µm and rotated ca. 230° (E) and 320° (F). Note that a thread stems from a tubulin ring on the equator of the gamete. Finer projections are also stained with dye.
(TIF)

Figure S10 **Approach of male gamete to three eggs and fertilization in** ***Pseudostaurosira trainorii*** **.** Time lapse LM. Scale  =  5 µm. A male gamete moves toward eggs. Note that the male gamete selects an eggs: the first two eggs are not fertilized even though the cell surfaces come in contact.
(TIF)

Figure S11 **Male gametes attach to female immature gametangia in** ***Pseudostaurosira trainorii*** **.** Time lapse LM. Scale  =  5 µm. Three male gametes are attached to a female chain which contains immature egg cells. The males wait for maturation of eggs in the female chain, and fertilize them as soon as the eggs mature. Male gametes and egg cells are marked m and e, respectively.
(TIF)

Figure S12 **Interrupted approach of a male gamete in** ***Pseudostaurosira trainorii*** **.** Time lapse LM. Scale  =  5 µm. A male gamete (bottom) approaches an egg cell, which is then fertilized by another male (top). The bottom male gamete does not head toward the zygote anymore, but instead starts wandering around the zygote. Male gametes and egg cells are marked m and e, respectively.
(TIF)

Figure S13 **Tests for mucilage around gametes and zygote in** ***Pseudostaurosira trainorii*** **.** LM with India Ink (A–F) and SEM (G–I). Scales = 5 µm (F, G) or 2 µm (H). **A**–**C.** No mucilage envelope is seen on egg (A), two male gametes attaching to egg (B) nor early stage of zygote (C). **D**–**F.** Globular mucilage envelopes are seen around expanding zygotes (auxospores). Note that the mucilage envelope keeps its globular shape while the auxospore expands bipolarly (F). **G.** Female clones with expanded eggs. No mucilage is seen. **H.** Zygote with mucilage envelope which has collapsed during drying. **I.** Enlarged view of H, showing fine fibres of mucilage.
(TIF)

Figure S14 **Result of pheromone experiment in** ***Pseudostaurosira trainorii*** **.** LM (A–C) and time lapse LM (D, E). Scales  =  50 µm (A–C) or 10 µm (D, E). **A, B.** Male (A) and female (B) partitions in Expt. 2 are filled with released gametes. **C.** Male gametes mixed with a vegetative female clone in Expt. 6. Male gametes are not attracted by a chain of vegetative female cells. **D.** In Expt. 7 male gametes are released but stay around their gametangia, rather than heading toward the gel (right side hyaline area marked with *) which is formed with the filtrate of sexualized female clone. **E.** Expt. 9 demonstrates that the dead eggs do not attract the male gametes. Note some male gametes attach to the eggs eventually leave: that has never happened with living eggs.
(TIF)

Figure S15 **Schematic illustrations of pheromone experiments showing male and female cells (blue and red dots, respectively), culture medium (light blue) and agar gel (light yellow).** **A.** Expt. 2. Compatible clones in separate holes in an agar plate. **B.** Expt. 6. Male clone incubated with a block of agar gel containing filtrate of female vegetative clone. **C.** Expt. 8. Compatible clones are confined to separate partitions but share the same medium.
(TIF)

Movie S1 **Male gamete in** ***Pseudostaurosira trainorii*** **retrieving the thread.** Real-time movie from which Fig. 2 is composed. Cell spins at on its axis, remaining in the same place. The vacuole seems to be elastic as the shape is changed by the thread tension.
(MOV)

Movie S2 **Male gamete in** ***Pseudostaurosira trainorii*** **with a blob.** Real-time movie from which Fig. 3 is composed. The gamete retrieves the thread and then extrudes a blob with cell elongation. The blob is retrieved by the spinning gamete. Finer projections show somewhat autonomous movement.
(MOV)
