# Supplementary figures and images for: Novel Sex Cells and Evidence for Sex Pheromones in Diatoms
Source: PLoS One. 2011 Oct 26;6(10):e26923. doi: 10.1371/journal.pone.0026923 (PMC3202595; doi:10.1371/journal.pone.0026923)

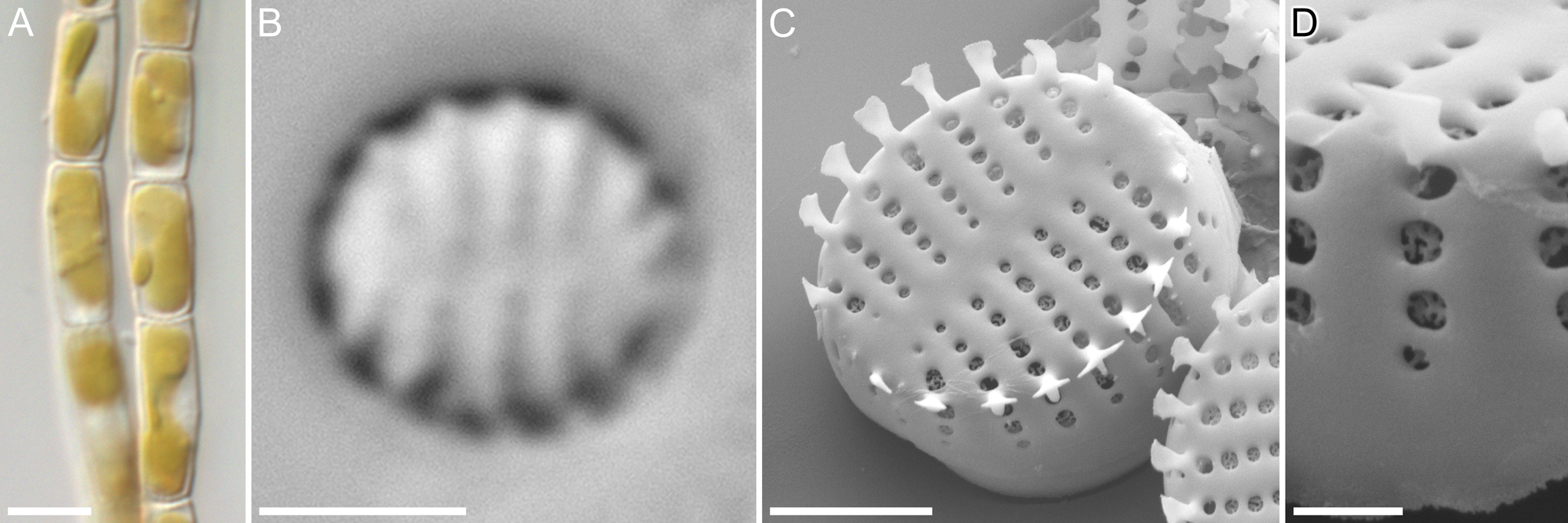

Supplement: Figure S1 — Vegetative cell in Pseudostaurosira trainorii. LM (A, B) and SEM (C, D). Scales = 5 µm (A), 2 µm (B, C) or 0.5 µm (D). A. Living cells attaching to each other to form a ribbon colony. B. Acid-cleaned valve showing parallel striae. C. Striae consisting of single rows of areolae. Valve with marginal spines. D. Enlargement of areolae, which are occluded by complex vela. (TIF) [file pone.0026923.s001.tif]

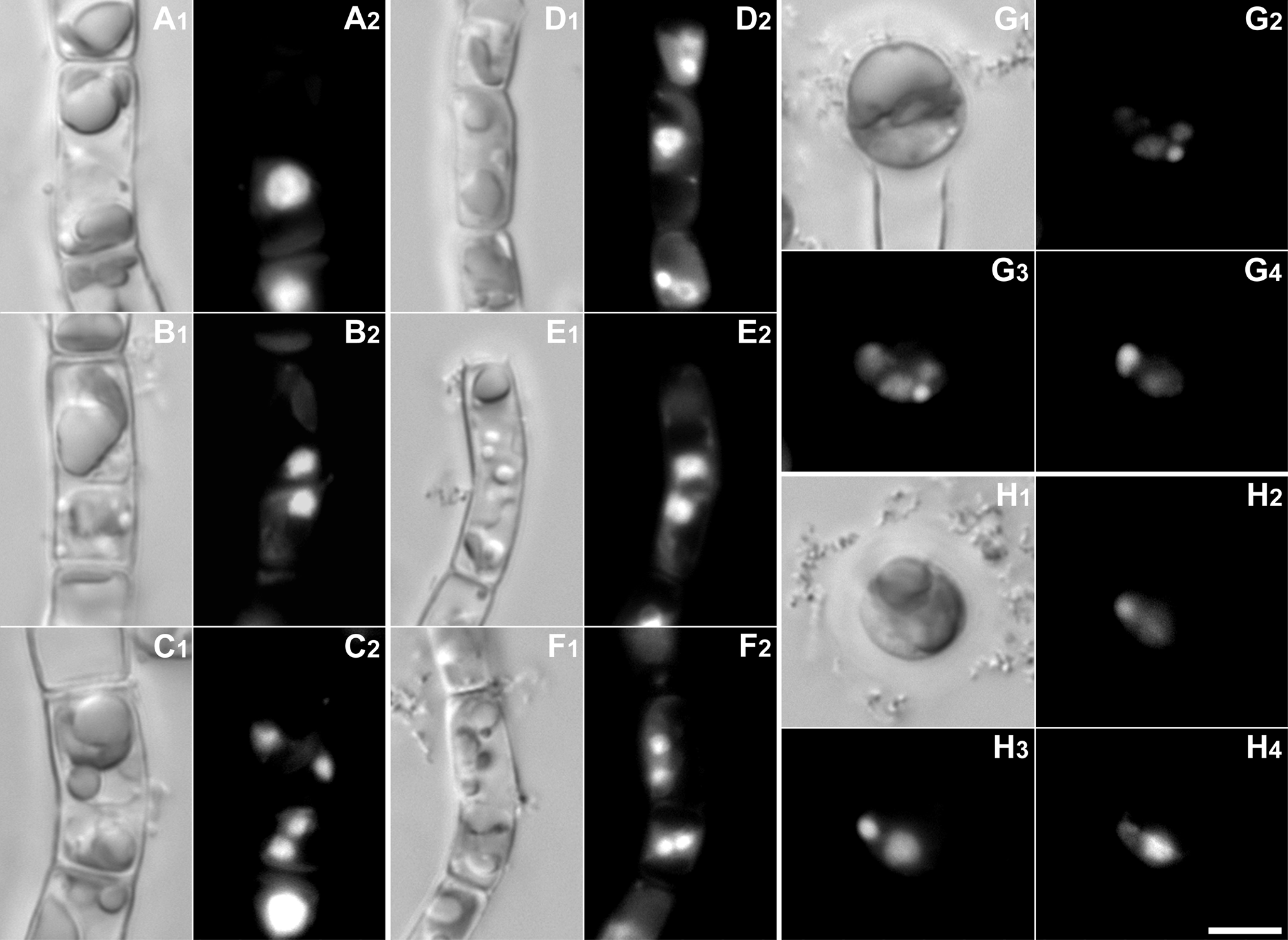

Supplement: Figure S2 — Nuclear behaviour in Pseudostaurosira trainorii visualized with DAPI. LM. Scale = 5 µm. A–C. Male clones. D–F. Female clones. G, H. Zygote. Bright field (1) and fluorescence image (2). A. Large nucleus at the centre, plastids appressed to valve. B. One nucleus per gamete after meiosis I. C. Meiosis II results in two nuclei per gamete. D. A large nucleus at the centre. E. Meiosis I results in two nuclei in a gametangium. F. Further nuclear division at meiosis II. Cytokinesis does not occur at meiosis II, resulting in four nuclei per gametangium, two in each gamete. G, H. Zygote observed under different focuses. The early stage shown in G lacks a mucilage envelope and contains four nuclei, two paler than the others; at a slightly later stage (H), the zygote is covered by a mucilage envelope and contains only two nuclei. (TIF) [file pone.0026923.s002.tif]

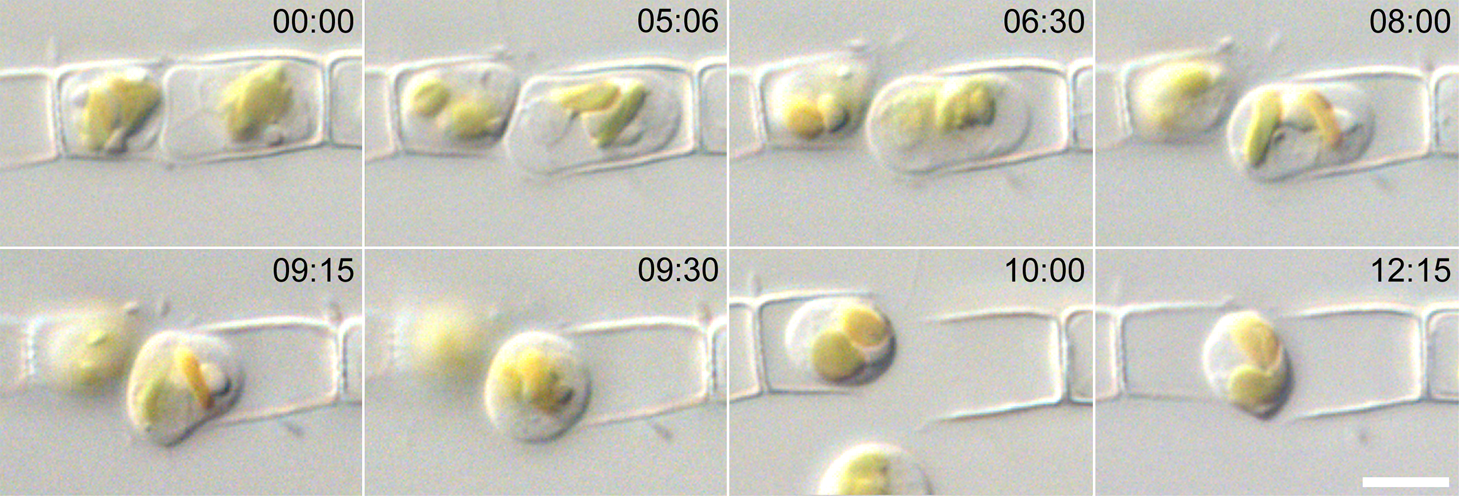

Supplement: Figure S3 — Male gamete liberation in Pseudostaurosira trainorii . Time lapse LM. Scale = 5 µm. The two gametes are unequal in size. The larger gamete (right) swells out from the gametangium. (TIF) [file pone.0026923.s003.tif]

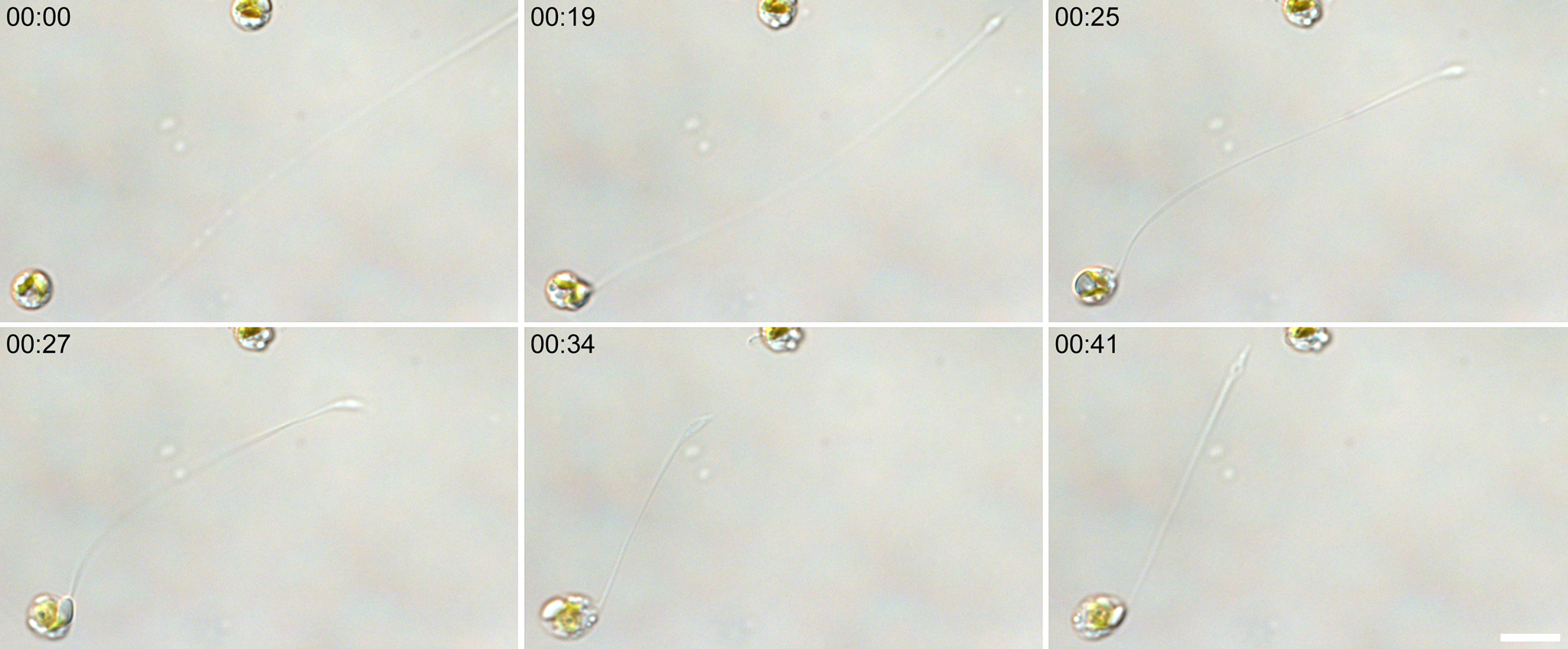

Supplement: Figure S4 — Retrieval and extrusion of thread of male gamete in Pseudostaurosira trainorii . Time lapse LM. Scale = 10 µm. A long thread is retrieved as the gamete spins, and then extruded again unless the thread is fully wound. (TIF) [file pone.0026923.s004.tif]

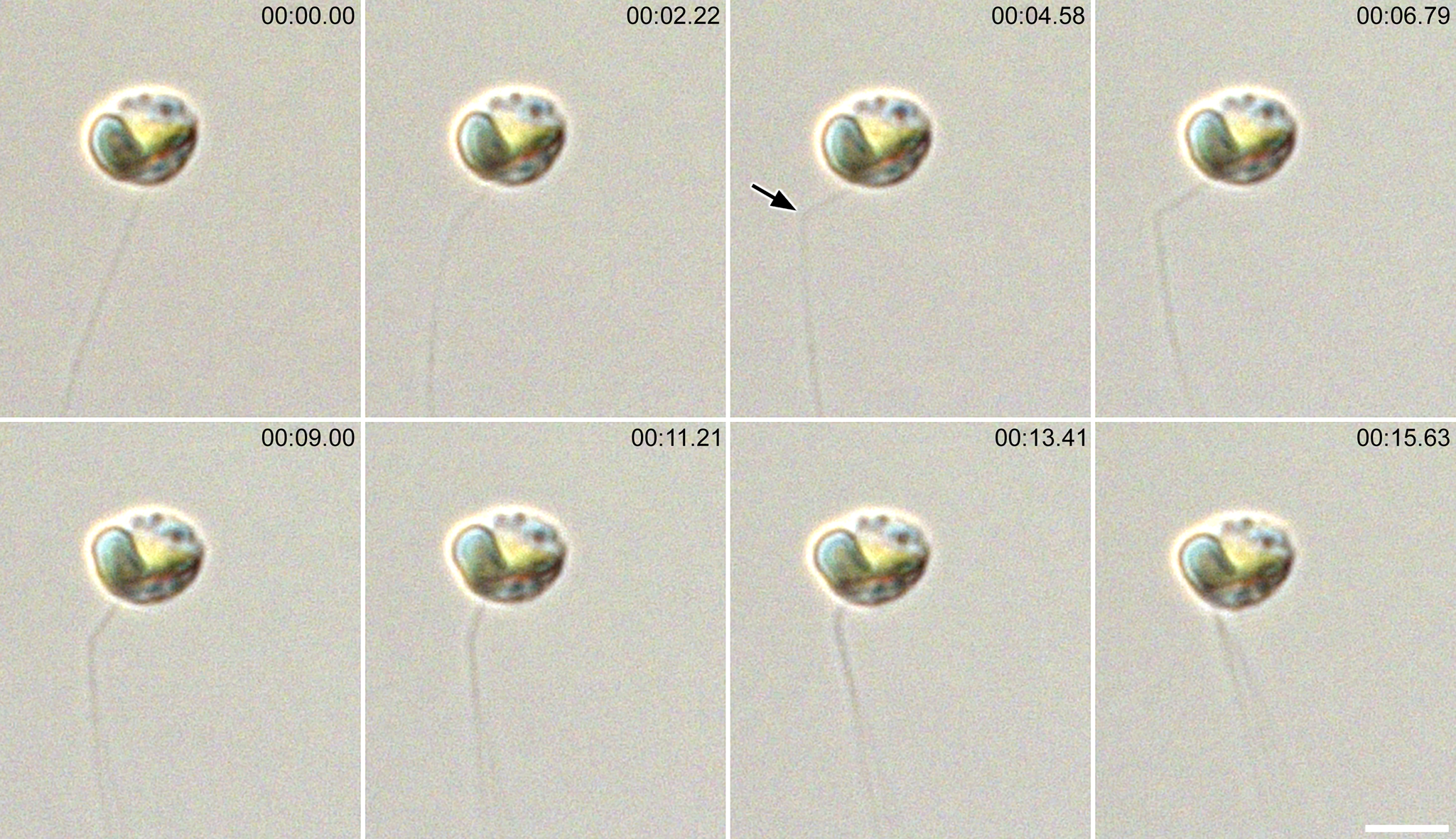

Supplement: Figure S5 — Autonomously folded thread on male gamete in Pseudostaurosira trainorii . Time lapse LM. Scale = 5 µm. Note the thread is folded near to the root from 00:04.58 onwards. (TIF) [file pone.0026923.s005.tif]

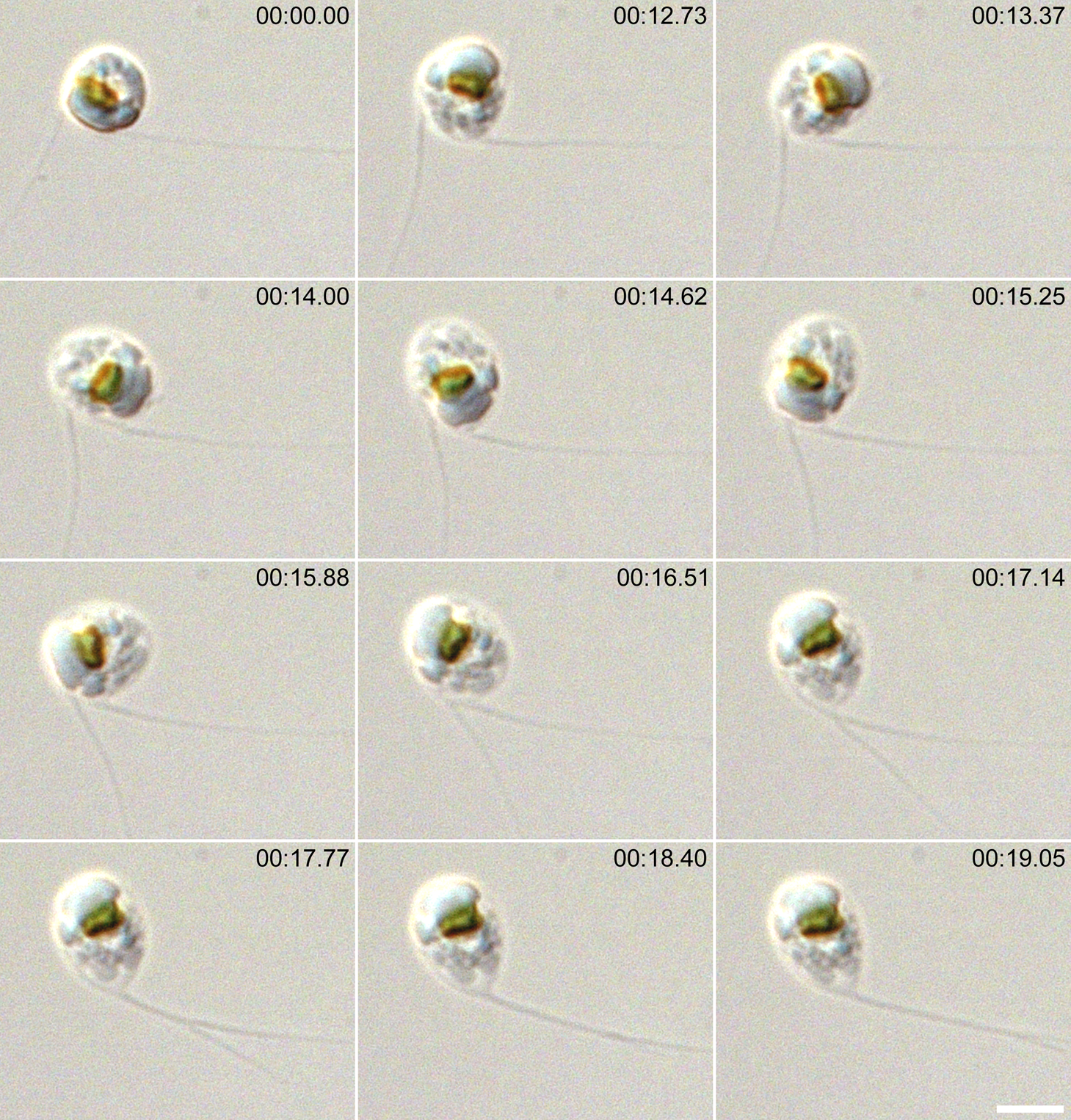

Supplement: Figure S6 — Fusing threads on male gamete in Pseudostaurosira trainorii . Time lapse LM. Scale = 5 µm. Gamete spins to retrieve threads (until 00:15.88), which are then fused from proximal end (00:16.51 onwards). Note the gamete does not spin when the threads are fusing. (TIF) [file pone.0026923.s006.tif]

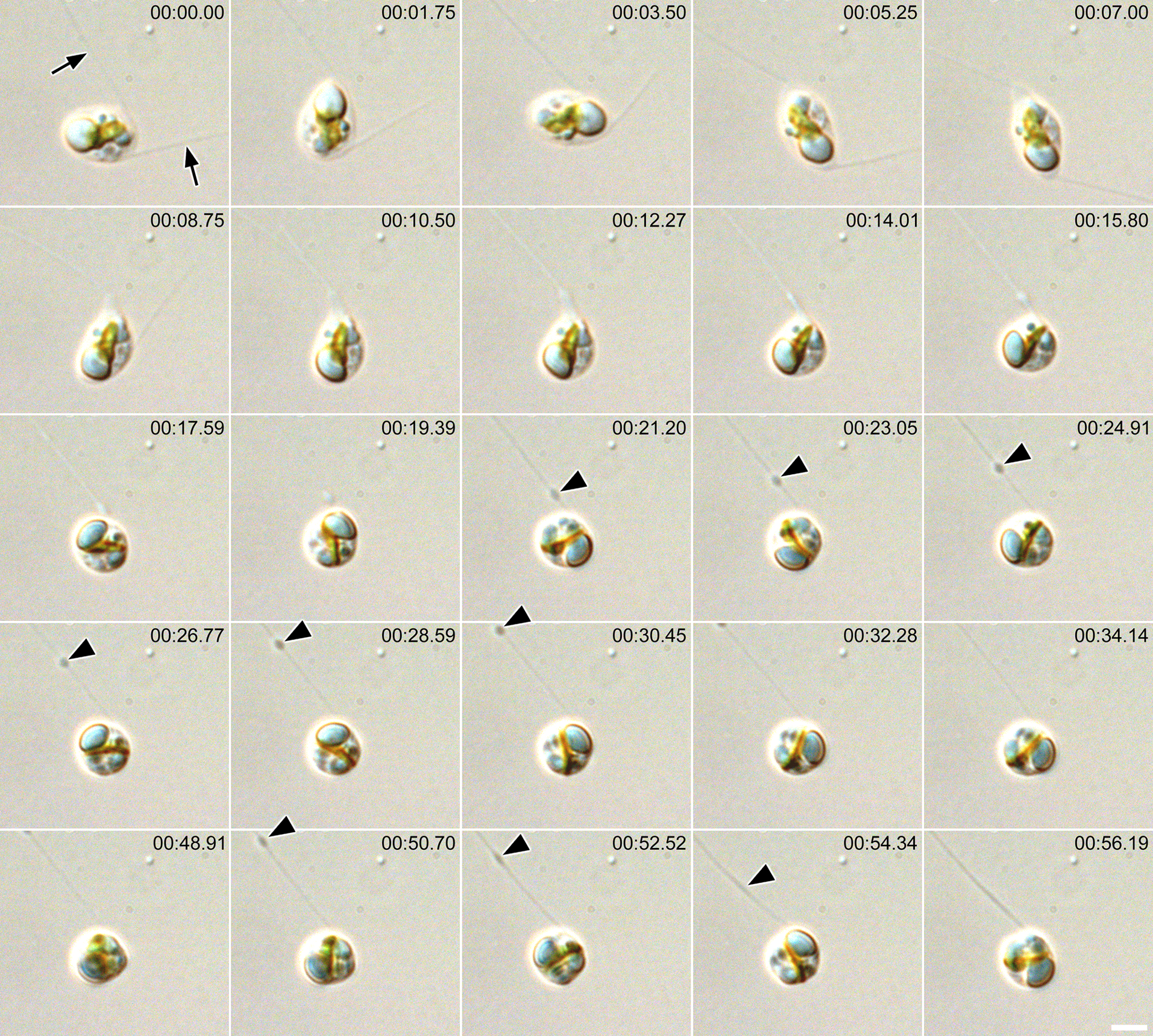

Supplement: Figure S7 — Thread behaviour of male gamete in Pseudostaurosira trainorii. Time lapse LM. Scale = 5 µm. When two threads fuse, a condensate (arrowhead) is formed at the bottom of the threads. The condensate moves distally as the gamete extrudes the thread, and then it moves back again proximally as the gamete retrieves the thread. Note that the shape of the condensate is somewhat globular when the thread is extruded, whereas it elongates and disappears at the end when the thread is retrieved. (TIF) [file pone.0026923.s007.tif]

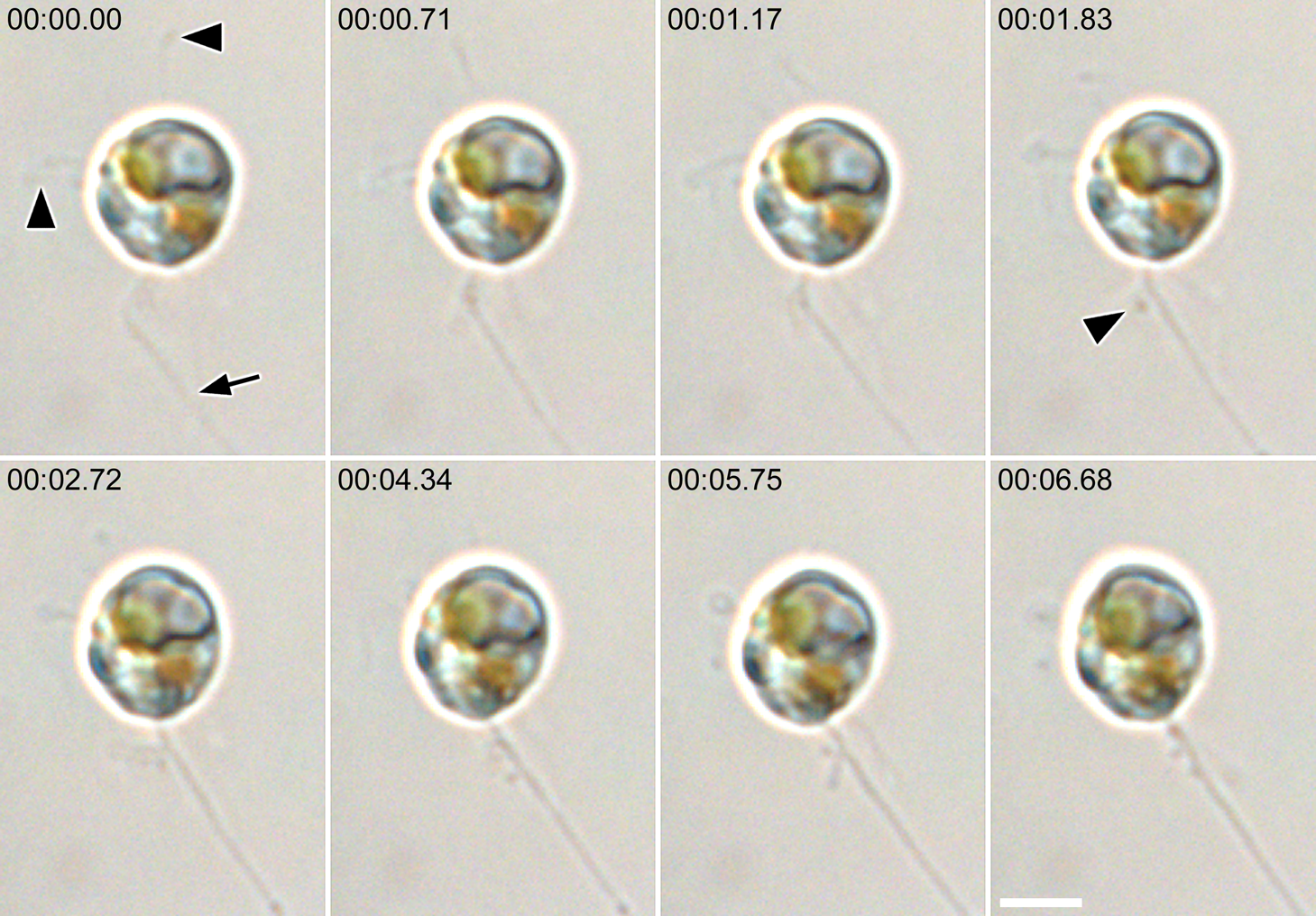

Supplement: Figure S8 — Finer projections of male gamete in Pseudostaurosira trainorii. Time lapse LM. Scale = 5 µm. Finer projections are seen on the gametic surface as well as on the thread. Arrow and arrowhead indicate thread and finer projections, respectively. (TIF) [file pone.0026923.s008.tif]

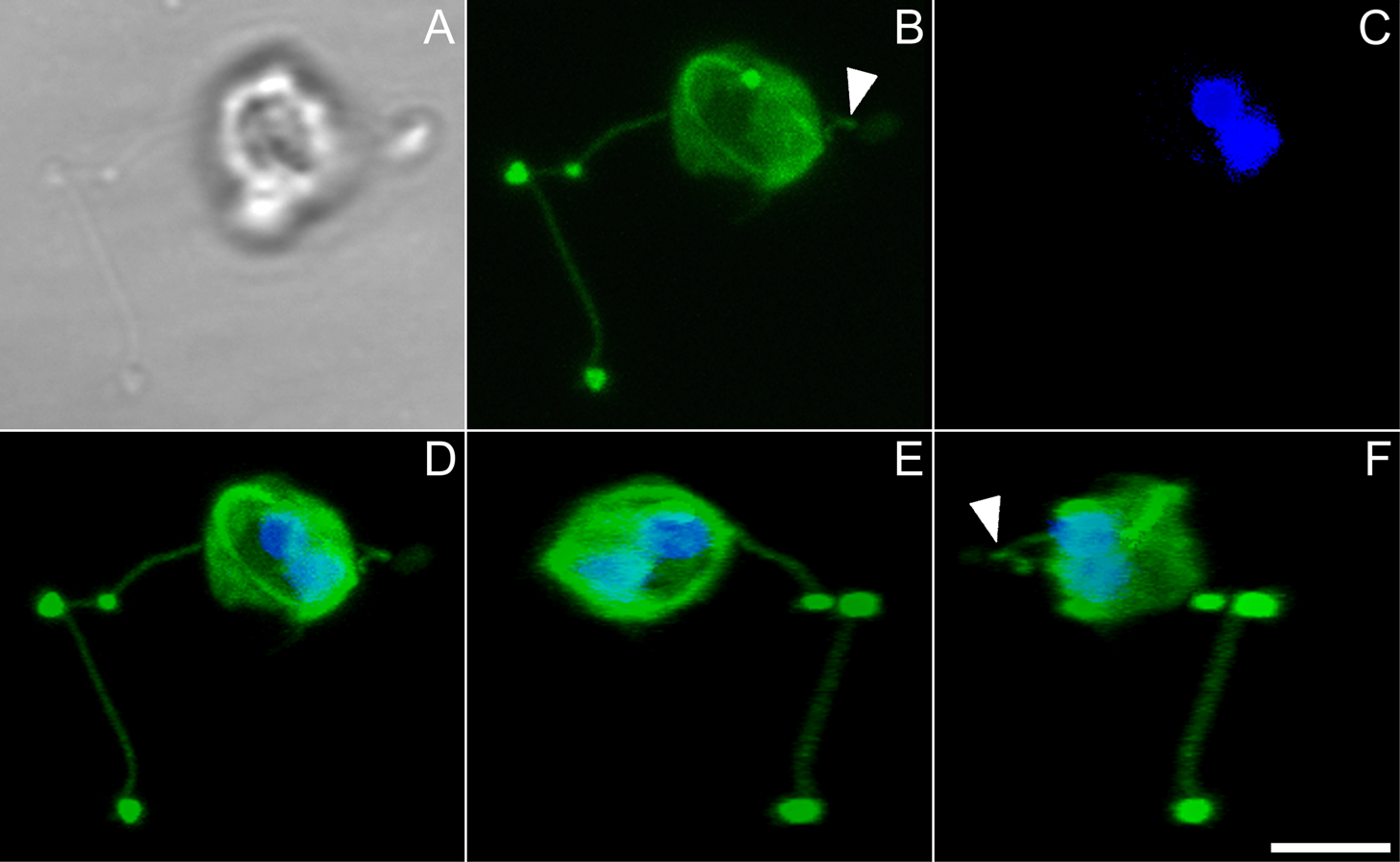

Supplement: Figure S9 — Male gamete extruding a thread. LM. Scale = 5 µm. A. Bright field optics. B. Tubulin immunolocalization. C. DNA staining with DAPI. D. Merged image of B and C. E, F. Three-dimensional reconstruction based on 27 optical stacks taken every 0.21 µm and rotated ca. 230° (E) and 320° (F). Note that a thread stems from a tubulin ring on the equator of the gamete. Finer projections are also stained with dye. (TIF) [file pone.0026923.s009.tif]

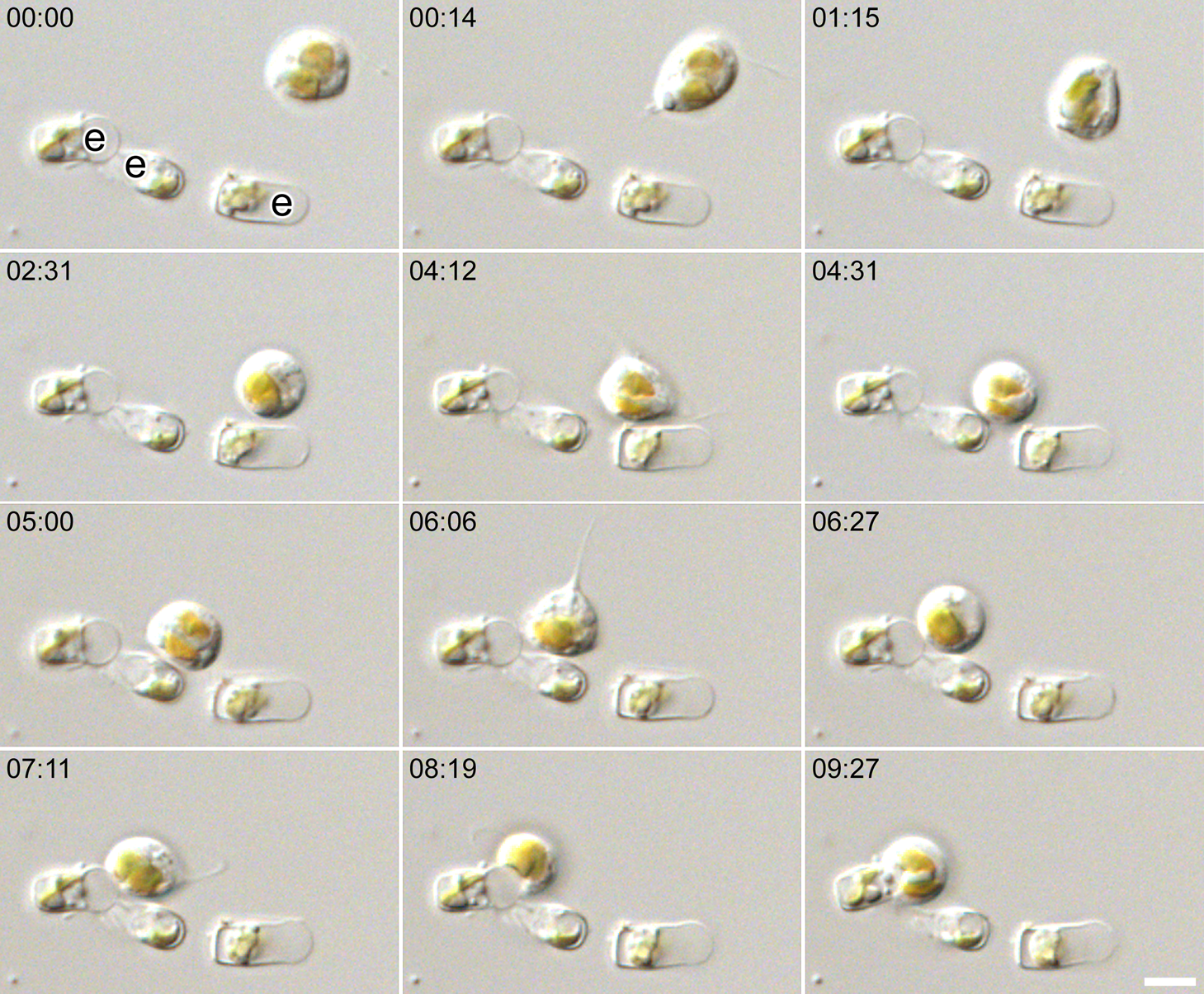

Supplement: Figure S10 — Approach of male gamete to three eggs and fertilization in Pseudostaurosira trainorii . Time lapse LM. Scale = 5 µm. A male gamete moves toward eggs. Note that the male gamete selects an eggs: the first two eggs are not fertilized even though the cell surfaces come in contact. (TIF) [file pone.0026923.s010.tif]

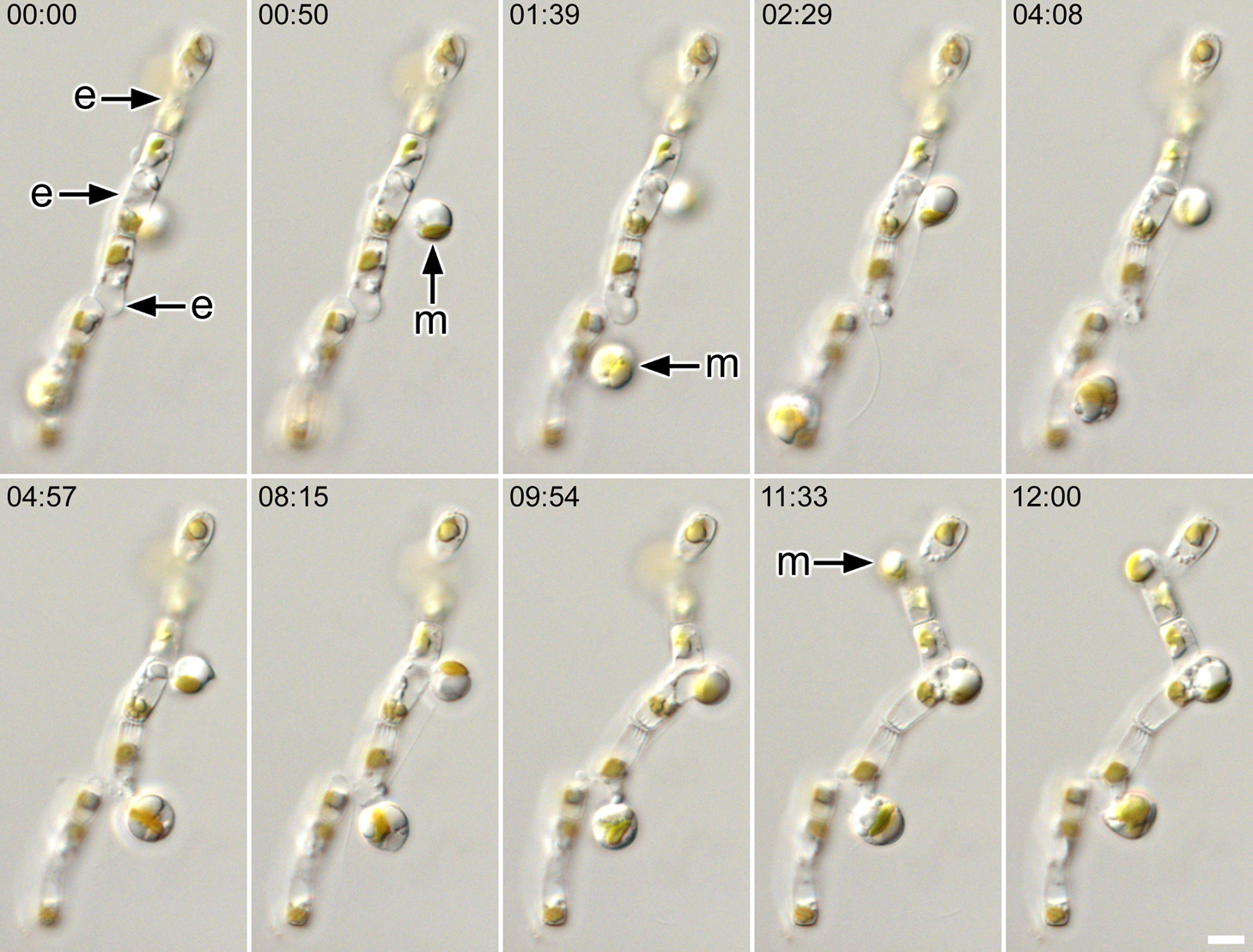

Supplement: Figure S11 — Male gametes attach to female immature gametangia in Pseudostaurosira trainorii . Time lapse LM. Scale = 5 µm. Three male gametes are attached to a female chain which contains immature egg cells. The males wait for maturation of eggs in the female chain, and fertilize them as soon as the eggs mature. Male gametes and egg cells are marked m and e, respectively. (TIF) [file pone.0026923.s011.tif]

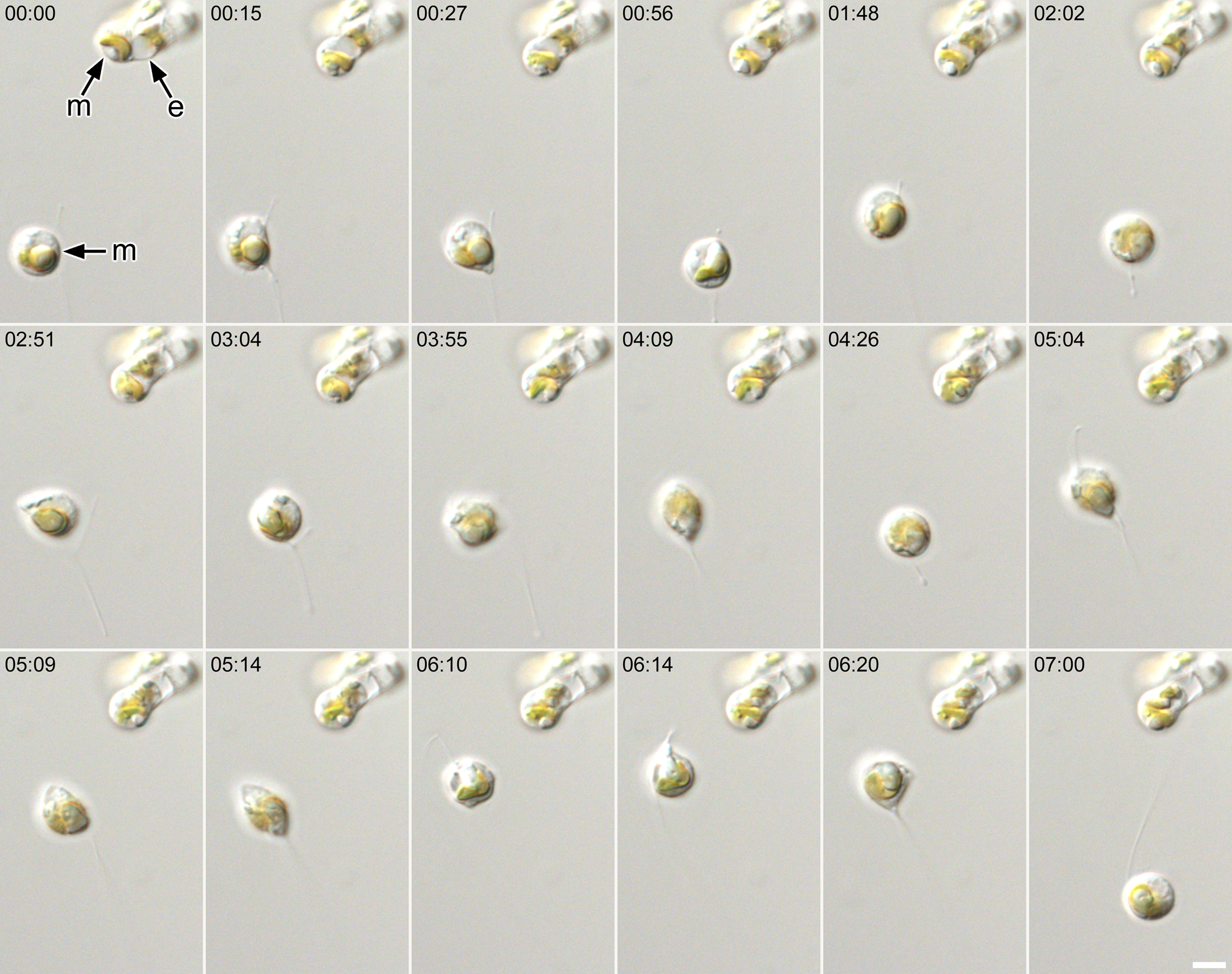

Supplement: Figure S12 — Interrupted approach of a male gamete in Pseudostaurosira trainorii . Time lapse LM. Scale = 5 µm. A male gamete (bottom) approaches an egg cell, which is then fertilized by another male (top). The bottom male gamete does not head toward the zygote anymore, but instead starts wandering around the zygote. Male gametes and egg cells are marked m and e, respectively. (TIF) [file pone.0026923.s012.tif]

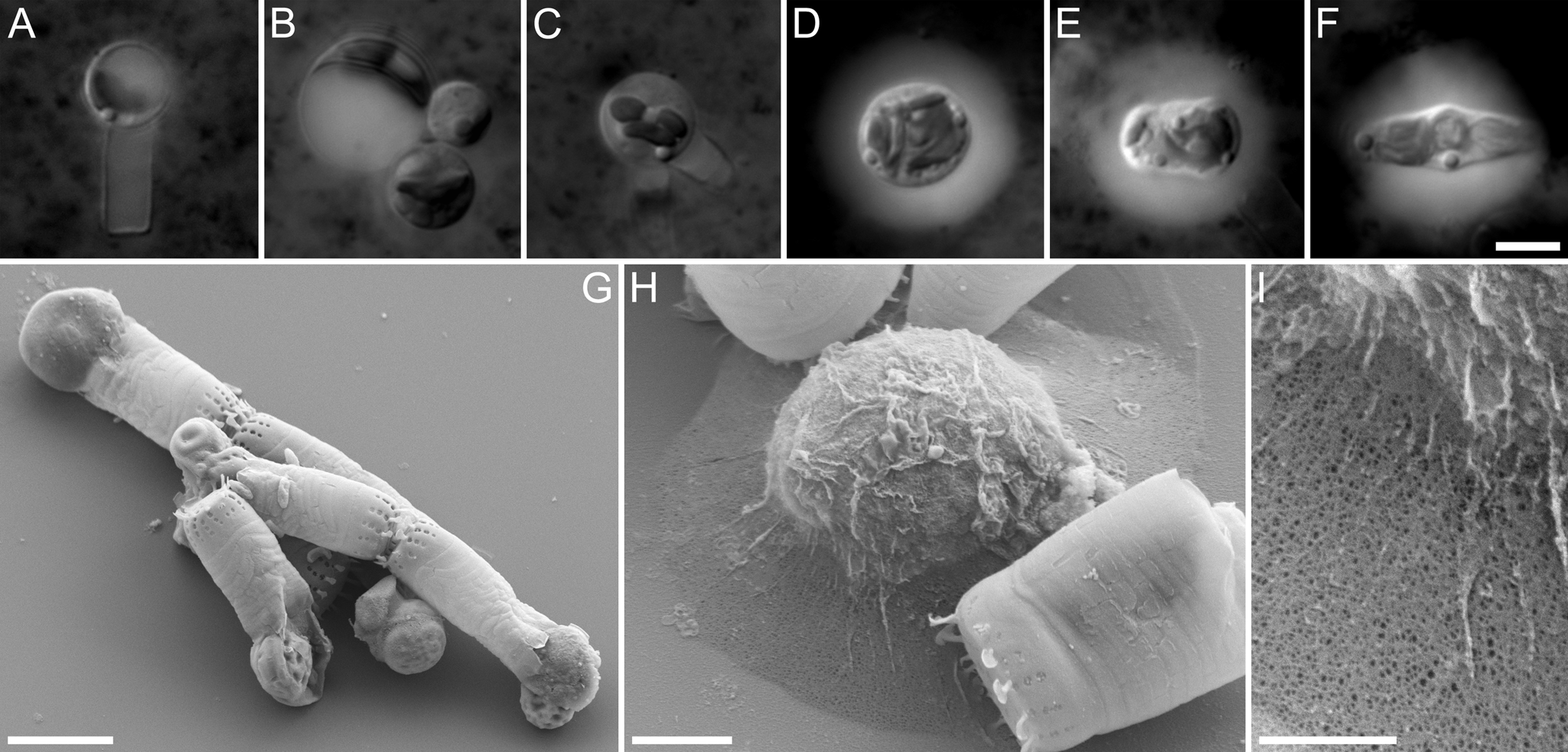

Supplement: Figure S13 — Tests for mucilage around gametes and zygote in Pseudostaurosira trainorii . LM with India Ink (A–F) and SEM (G–I). Scales = 5 µm (F, G) or 2 µm (H). A–C. No mucilage envelope is seen on egg (A), two male gametes attaching to egg (B) nor early stage of zygote (C). D–F. Globular mucilage envelopes are seen around expanding zygotes (auxospores). Note that the mucilage envelope keeps its globular shape while the auxospore expands bipolarly (F). G. Female clones with expanded eggs. No mucilage is seen. H. Zygote with mucilage envelope which has collapsed during drying. I. Enlarged view of H, showing fine fibres of mucilage. (TIF) [file pone.0026923.s013.tif]

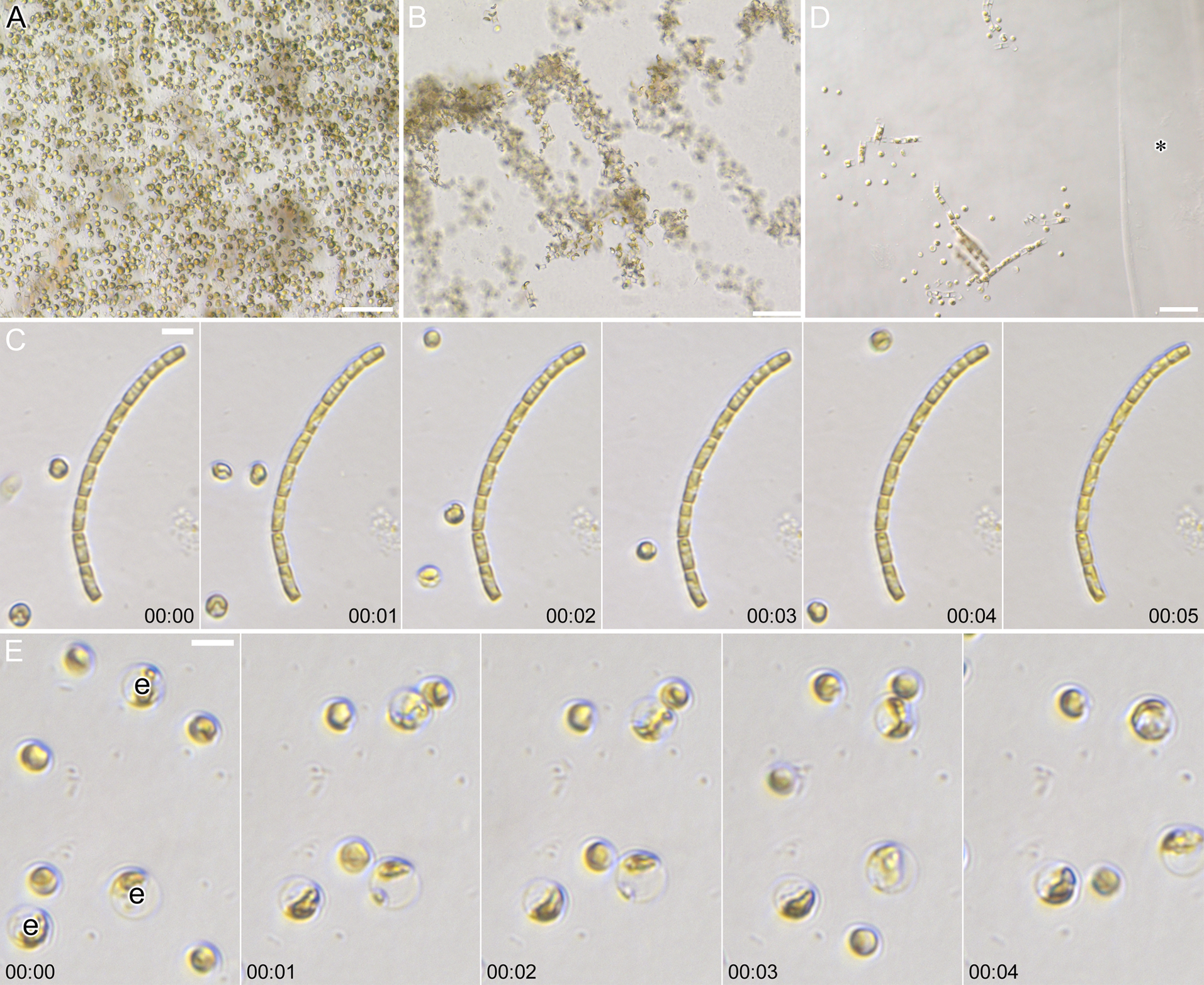

Supplement: Figure S14 — Result of pheromone experiment in Pseudostaurosira trainorii . LM (A–C) and time lapse LM (D, E). Scales = 50 µm (A–C) or 10 µm (D, E). A, B. Male (A) and female (B) partitions in Expt. 2 are filled with released gametes. C. Male gametes mixed with a vegetative female clone in Expt. 6. Male gametes are not attracted by a chain of vegetative female cells. D. In Expt. 7 male gametes are released but stay around their gametangia, rather than heading toward the gel (right side hyaline area marked with *) which is formed with the filtrate of sexualized female clone. E. Expt. 9 demonstrates that the dead eggs do not attract the male gametes. Note some male gametes attach to the eggs eventually leave: that has never happened with living eggs. (TIF) [file pone.0026923.s014.tif]

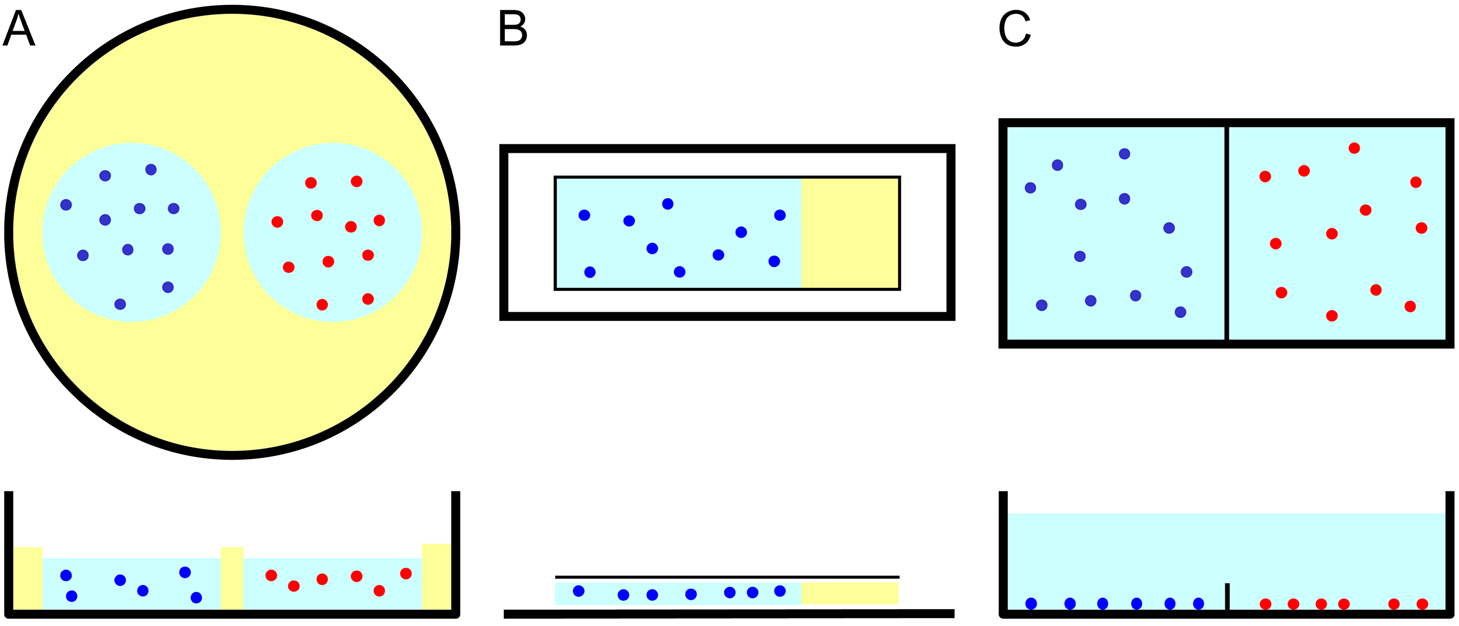

Supplement: Figure S15 — Schematic illustrations of pheromone experiments showing male and female cells (blue and red dots, respectively), culture medium (light blue) and agar gel (light yellow). A. Expt. 2. Compatible clones in separate holes in an agar plate. B. Expt. 6. Male clone incubated with a block of agar gel containing filtrate of female vegetative clone. C. Expt. 8. Compatible clones are confined to separate partitions but share the same medium. (TIF) [file pone.0026923.s015.tif]
